# Biofilm lifestyle across different lineages of ammonia-oxidizing archaea

**DOI:** 10.1093/ismejo/wraf182

**Published:** 2025-09-09

**Authors:** Maximilian Dreer, Thomas Pribasnig, Logan H Hodgskiss, Zhen-Hao Luo, Fran Pozaric, Christa Schleper

**Affiliations:** Archaea Biology and Ecogenomics Unit, Department of Functional and Evolutionary Ecology, University of Vienna, Djerassiplatz 1, Vienna 1030, Austria; Archaea Biology and Ecogenomics Unit, Department of Functional and Evolutionary Ecology, University of Vienna, Djerassiplatz 1, Vienna 1030, Austria; Archaea Biology and Ecogenomics Unit, Department of Functional and Evolutionary Ecology, University of Vienna, Djerassiplatz 1, Vienna 1030, Austria; Archaea Biology and Ecogenomics Unit, Department of Functional and Evolutionary Ecology, University of Vienna, Djerassiplatz 1, Vienna 1030, Austria; Archaea Biology and Ecogenomics Unit, Department of Functional and Evolutionary Ecology, University of Vienna, Djerassiplatz 1, Vienna 1030, Austria; Archaea Biology and Ecogenomics Unit, Department of Functional and Evolutionary Ecology, University of Vienna, Djerassiplatz 1, Vienna 1030, Austria

**Keywords:** biofilms, nitrogen cycle, archaea, ammonia-oxidizing archaea, nitrification, multicopper oxidase

## Abstract

Although ammonia-oxidizing archaea (AOA) are globally distributed in nature, growth in biofilms has been relatively little explored. Here, we investigated six representatives of three different terrestrial and marine clades of AOA in a longitudinal and quantitative study for their ability to form biofilm, and studied gene expression patterns of three representatives. Although all strains grew on a solid surface, soil strains of the genera *Nitrosocosmicus* and *Nitrososphaera* exhibited the highest capacity for biofilm formation. Based on microscopic and gene expression data, two different colonization strategies could be distinguished. S-layer containing AOA (from both soil and marine habitats) initialized attachment as single cells, subsequently forming denser layers, whereas the S-layer free species of the *Nitrosocosmicus* clade attached as suspended aggregates to the surface and henceforth showed fastest establishment of biofilm. Transcription profiles were significantly different between planktonic and biofilm growth in all strains, and revealed individual transcriptomic responses, albeit fulfilling shared functions. In particular, the strong expression of different types of multicopper oxidases was observed in all strains suggesting modifications of their cell coats. S-layer carrying AOA each additionally expressed a set of adhesion proteins supporting attachment. Detoxification of nitrous compounds, copper acquisition as well as the expression of transcription factor B were also shared responses among biofilm producing strains. However, the majority of differentially expressed protein families was distinct among the three strains, illustrating that individual solutions have evolved for the shared growth mode of biofilm formation in AOA, probably driven by the different ecological niches.

## Introduction

Nitrification, the microbially mediated oxidation of ammonia (NH_3_) to nitrate (NO_3_^−^) via nitrite (NO_2_^−^), is a key process of the global biogeochemical nitrogen cycle. The first and rate-limiting step of ammonia oxidation (NH_3_ to NO_2_^−^) is performed by ammonia-oxidizing archaea (AOA) [1, 2], ammonia-oxidizing bacteria (AOB) [3], and the recently discovered complete ammonia-oxidizing bacteria capable of complete oxidation of NH_3_ to NO_3_^−^ [4, 5]. All ammonia-oxidizing microorganisms contribute directly or indirectly to the production of nitrous oxide [6, 7], a potent greenhouse gas, and to the loss of nitrogen in natural and in fertilized agricultural systems [8, 9]. It is therefore of continuous importance to better understand their metabolisms and activities in diverse ecosystems. AOA are ubiquitous in most aerobic environments, ranging from the sediments of the Mariana trench [10] to the soils of Mount Everest [11]. They outnumber their bacterial counterparts in most oligotrophic environments including pristine soils and the open ocean [12, 13], often by orders of magnitude. Although physiological studies have indicated a specialization to low nutrient environments [14, 15], AOA populations additionally outnumber AOB in highly fertilized agricultural soils [12].

Biofilms have been shown to be a widespread growth mode of bacteria and archaea. It has been hypothesized that 40%–80% of cells on earth form or are part of biofilms in-situ, driving biogeochemical cycles [16]. Whereas most soil microorganisms are thought to grow in biofilms, marine organisms can grow either planktonically or particle associated, offering specialized niches for different species [17]. Although most of the isolated AOA strains from aquatic or terrestrial environments have been isolated in liquid media [1, 18], some species from soil, in particular those of the *Nitrosocosmicus* clade (Genome Taxonomy Database [19] used throughout) were reported to grow on solid surfaces [20–22], or as aggregates in liquid culture [23], indicating their capacity to grow in biofilms. Additionally, genomic analysis indicated the capacity of all *Nitrososphaeraceae* and to a lesser extent *Nitrosopumilaceae* for production of extracellular polymeric substances (EPS) and cell surface modifications, hallmarks of biofilm formation [24]. Regardless of these observations, research on biofilm-associated AOA and especially the capability of AOA to form biofilms is scarce and mostly circumstantial. Even though AOA have been found to be part of biofilms in pristine and manmade environments like hot springs [25, 26] and wastewater treatment plants [27, 28], studies on environmental samples, or enrichments from biofilms commonly focus on the abundance of AOA in mixed communities and their contribution to nitrification rather than studying the biofilm phenotype. Nevertheless, isolated *Nitrosocosmicus* species were shown to grow as aggregates in liquid culture, display putative extracellular polymeric substances, and attach to surfaces, with Ca. Nitrosocosmicus oleophilus MY3 displaying increased growth rates upon attachment [20, 23]. Outside of the genus *Nitrosocosmicus*, *Nitrososphaera viennensis* has also been observed to exhibit biofilm growth on different glass surfaces [24, 29]. However, a thorough investigation giving insight into the capability of biofilm formation of different AOA clades is lacking, as is a fundamental understanding of their physiology under these conditions.

We hypothesize that biofilm formation is a trait found in different lineages across the diversity of AOA and that conserved physiological changes, including EPS production and cell surface modification, can be identified in this potentially ecologically relevant mode of growth. The propensity of six representatives, covering major lineages of AOA, to form biofilm was studied and determined by a growth assay using borosilicate glass as a surface. Strains included were AOA isolated from garden, arable, and acidic arable soil (*Nitrososphaera viennensis* EN76, Ca. *Nitrosocosmicus franklandianus* C13, and *Nitrosotalea sinensis* Nd2, respectively), tropical marine aquarium gravel (*Nitrosopumilus maritimus* SCM1), and coastal surface water (*Nitrosopumilus piranensis* D3C*, Nitrosopumilus adriaticus* NF5) [1, 18, 23, 30, 31]. A genome wide transcriptomic analysis comparing planktonic growth to growth in a biofilm of a representative of each of three major lineages of AOA, *N. viennensis*, Ca. N. franklandianus (hereafter referred to as *N. franklandianus*), and *N. maritimus* was performed and is presented along with a comparison of conserved protein families of the biofilm phenotype and an AOA-wide genomic analysis for identified bona fide biofilm genes. Additionally, scanning electron microscopy (SEM) and light microscopy imaging of a time series elucidates the architecture and succession of AOA biofilms in individual strains.

## Materials and methods

Detailed cultivation description, culture maintenance, protocols for RNA extraction, transcriptomic analysis, and phylogenetic tree calculation are available in the supplementary material.

### Growth as biofilm

Biofilms are defined by the International Union of Pure and Applied Chemistry (IUPAC) as “aggregates of microorganisms in which cells are frequently embedded in a self-produced matrix of extracellular polymeric substances (EPS) that are adherent to each other and/or a surface” [32]. To achieve AOA biofilm growth, two different surfaces were used. Borosilicate cover glasses (CG) (VWR, 631–0120, 18 × 18 mm) or soda-lime glass microscope slides (MS) with a salinized surface leading to a positive charge (Carl Roth, Histobond, CEX0.1, 76 × 26 × 1 mm) were sterilized by autoclaving in Milli-Q water, after which they were individually added to 30 ml polystyrene containers or 250 ml Schott bottles containing 20 ml or 125 ml growth media respectively using tweezers sterilized with Incidin. The surface area for biofilm formation was scaled up from CG to MS after RNA yields for sequencing were below needed thresholds (Supplementary methods, Fig. S1). The growth media were inoculated and strains grown as described in supplementary material and summarized in Table S1. Upon reaching a predefined range of nitrite (Table S1), the CG or MS were transferred to fresh growth media using sterilized tweezers. To ensure that only cells attached to the provided surfaces were transferred, excess liquid forming droplets at the edges of CG was absorbed using UV sterilized Whatman paper (Whatman GB003). MS were carefully dipped three times in prewarmed basal media before being transferred to fresh medium, as using Whatman paper was insufficient to remove all excess liquid (Supplementary methods, Dataset S1). Using this setup CG and MS were continuously transferred. Nitrite was measured and cultures checked for purity as described in supplementary materials for stock cultures. Biofilm cultures were never inverted before sampling to prevent the disruption of the biofilm. CG were fully submerged standing upright and slightly tilted at the bottom of the 30 ml containers due to the container’s internal diameter of 22.38 mm. MS were not fully submerged, the frosted area not being covered, standing upright and strongly tilted in 250 ml Schott bottles. After reaching the species-specific fastest nitrite production, five MS each of *N. viennensis*, *N. franklandianus*, and *N. maritimu*s biofilms were frozen on dry ice and stored at −70°C for RNA extraction.

To be able to quantitatively compare the biofilm-forming capabilities of different strains, a “biomass accumulation ratio” (BAR) was calculated*—*taking species-specific generation times into account. The time required to produce 500 μM of nitrite was determined for both planktonic 5% inoculation volume cultures and all CG transfers, and was termed standard time (ST) and biofilm time (BT), respectively. The BAR was then calculated by dividing the ST by the BT for each transfer, expressed as BAR = ST/BT_x_, where *x* represents the transfer number.

### Microscopy of biofilms

To directly image undisturbed biofilm on CG, imaging spacers from an adhesive sheet with a thickness of 0.12 mm (Grace Bio-Labs SecureSeal adhesive sheets) were prepared. The sheet was cut into CG sized frames with an inner dimension of ~13 x 13 mm, attached to microscopy slides and 40 μl of basal medium (FWM or SCM) was added to the middle of the frame. Active CG biofilms were immediately mounted to the prepared frames using tweezers after taking off excess liquid as described above. The CG were sealed with nail polish, dried for 15 minutes in the dark at room temperature, and imaged with phase contrast microscopy.

For scanning electron microscopy CG were prepared as follows. After initial in-situ fixation of CG in growth media with 2.5% glutaraldehyde for 5 min at room temperature, CG were transferred to PBS buffer containing 2.5% glutaraldehyde and stored at 4°C overnight. The CG were washed three times in PBS before being dehydrated in an ethanol series (30%, 50%, 70%, 80%, 90%, 100%) and dried via either critical point drying with CO_2_ (*N. viennensis*, *N. maritimus*), or chemically using hexamethyldisilazane (*N. franklandianus*). Dried samples were sputter coated with Au and imaged in a JEOL IT 300 scanning electron microscope at 20 kV. To prevent the disruption of biofilm structures CG were standing upright throughout the whole process using the 30 ml polystyrene containers for initial fixation, 50 ml Falcon tubes for fixation at 4°C and dehydration, and the cover glass holder of the Leica EM CPD300 for critical point drying.

## Results

### Biofilm formation in different AOA

**Figure 1:**
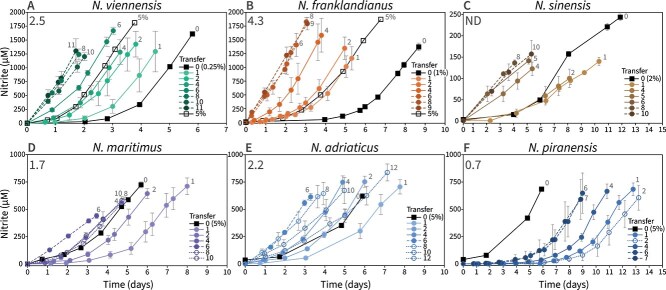
Biofilm formation of ammonia-oxidizing archaea on CG. Nitrite production of *Nitrososphaera viennensis* (A)*, Nitrosocosmicus franklandianus* (B), *Nitrosotenuis sinensis* (C), *Nitrosopumilus maritimus* (D), *Nitrosopumilus adriaticus* (E)*,* and *Nitrosopumilus piranensis* (F) grown as biofilm on CG. For illustrative purposes not all transfers are depicted. The raw data of all transfers can be found in Dataset S1. Initial planktonic growth of cells in the presence of CG (black lines, filled rectangles), continuous transfers of CG (colored lines, filled circles), and maximum nitrite production (colored dashed lines, filled circles) are shown. Drops in nitrite production between transfers were observed for marine strains (dotted lines, empty circles). Cultures started with 5% inoculation volume done in duplicate are shown for *N. viennensis* and *N. franklandianus* (black lines, empty rectangles). Maximum biomass accumulation ratios (BAR) values are displayed for each species (Table S2). Increasing numbers of transfers are indicated by numbers next to lines and darkening color gradients. Nitrite measurements show averages of eight biological replicates. Error bars depict the standard deviation.

AOA were initially grown planktonically in the presence of a borosilicate cover glass (CG) as a surface for attachment before consecutive transfers of only the CG and attached cells. The ability of AOA to attach to CG and accumulate biomass over time was investigated by following nitrite production, which is a well-established proxy for cell numbers [1, 18] (Fig. 1, Dataset S1). In this experimental setup, nitrite production did not exclusively stem from biofilms, as a planktonic fraction was observed for every transfer. However, only cells adhering to cover glass were transferred, implying that the planktonic fraction in the following cultures was seeded from biofilms. Faster nitrite accumulation should therefore represent an increased amount of biomass on the cover glass. The speed of nitrite accumulation of all tested AOA increased over the course of multiple CG transfers indicating cumulative increase in biofilm biomass.

The soil AOA *N. viennensis* and *N. franklandianus* exhibited a clear increase in nitrite production already after the initial cover glass transfer that continuously accelerated with each consecutive transfer (Fig. 1A and B). A stable maximum nitrite production for both species was reached after eight and six transfers respectively (Fig. 1A and B). Similarly, *N. sinensis* reached a stable maximum nitrite production after eight transfers, well above the nitrite production of initial growth. However, its growth was considerably slower than the two soil strains and not exponential. The nitrite production of all marine AOA strains was less stable with successive CG transfers indicating a less pronounced capability of biofilm formation (Fig. 1D–F). After peaking at the sixth transfer, nitrite production of *N. maritimus* decreased and stabilized at a lower level, whereas it steadily declined in *N. adriaticus*. Unlike the other marine strains, the nitrite production of *N. piranensis* decreased from transfer one to two, before showing an increase in nitrite production at consecutive transfers. Along with its high standard deviations in successive CG transfers and lowest BARs (for calculation see Materials and Methods), consistently <1, this suggests the lowest capability to stably adhere to surfaces as compared to all other strains (Fig. 1F, Table S2).

The differences between the initial growth and first transfer of the *Nitrososphaeraceae* and *Nitrosopumilaceae* species can be attributed to the different inoculation volumes used, ranging from 0.25% to 5% (volume/volume), which were chosen to grant cells of each species a similar amount of time to adhere to the provided surface in the initial inoculation. A line displaying growth of a 5% (v/v) inoculation culture was added for *N. viennensis* and *N. franklandianus* as reference (Fig. 1).

The maximum BARs support that *N. franklandianus* had the highest biofilm-forming capabilities. Although the marine strains *N. maritimus* and *N. adriaticus* initially displayed biofilm-forming abilities comparable to those of *N. viennensis*, they were not stable over time (Fig. 1 and Table S2). Because *N. sinensis* did not exhibit exponential growth and is limited in its nitrite production capacity in pure culture [33], it was excluded from the analysis.

### Time series of biofilm formation

**Figure 2:**
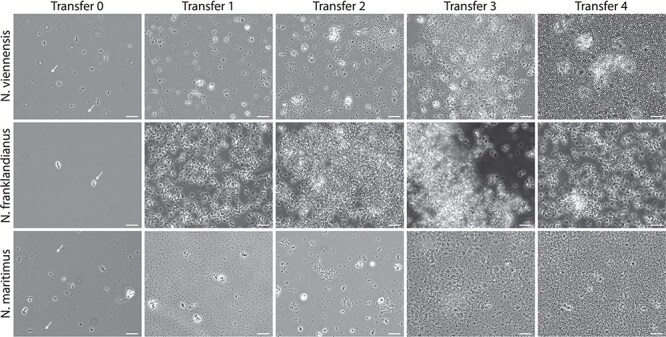
Light microscopy time series of *N. viennensis, N. franklandianus*, and *N. maritimus* in-situ biofilm formation on cover glass (CG). CG were destructively sampled over four transfers for light microscopy imaging after production of 1200-1600 μM NO_2_^−^. (Transfer 0) initial growth in presence of a CG. (Transfers 1–4) first to fourth transfer of CG. White arrows indicate cells initially adhering to a CG (Transfer 0). Observed features included: multicellular structures/microcolonies (Transfers 1–2). Three-dimensional, multilayered structures (*N. franklandianus* Transfer 2–4, others Transfer 3–4; features out of focus appeared white and blurry). Biofilm fully covering the CG (*N. franklandianus* transfer 2–3, others Transfer 4). Scale bars depict 10 μm.

Three species were chosen for further analysis due to their biofilm-forming potential and culturing feasibility: *N. viennensis*, *N. franklandianus*, and *N. maritimus*. Each was imaged for five consecutive transfers by light microscopy, destructively sampling a CG for each transfer (Fig. S1). After initial growth in the presence of CG, single or small aggregates of attached cells were observed (Fig. 2, Transfer 0). Over the course of the two following transfers, microcolonies and larger 3D structures formed and continued to expand (Fig. 2, Transfer 1–3). These structures eventually merged, establishing either multilayered, 3D biofilms in *N. viennensis* and *N. franklandianus*, or a monolayer with small interspersed aggregations in *N. maritimus* (Fig. 2, Transfer 2–3). The biofilm of all species eventually covered the whole surface of the CG in a continuous or interspersed layer (Fig. 2, Transfer 4).

Two differing colonization strategies were identified: *N. viennensis* and *N. maritimus* attached to the CG as single cells, which formed microcolonies that subsequently merged into more extensive 3D structures. In contrast, the attachment of *N. franklandianus* appeared to be based on the deposition of small, pre-formed suspended aggregates and happened much more rapidly, with 3D structures already forming from the second transfer on. Along with its rapid biofilm development, *N. franklandianus* also formed the most extensive, multilayered biofilms, characterized by the height and area of the observed 3D structures. (Fig. 2, *N. franklandianus*-Transfers 2 and 3).

### Different morphologies of AOA biofilms

**Figure 3:**
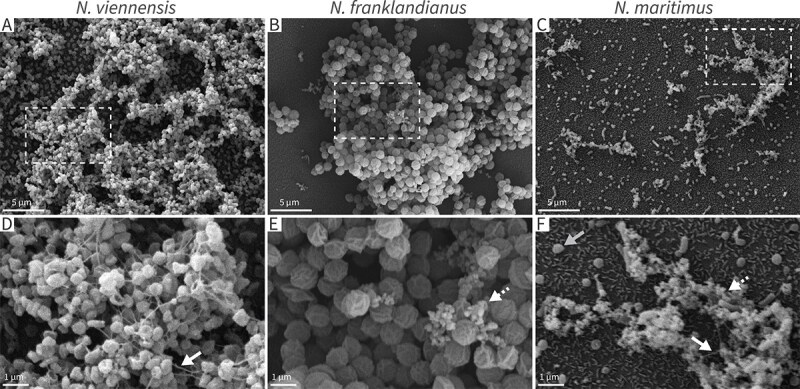
Visualization of AOA biofilms by SEM. *N. viennensis* (A, D), *N. franklandianus* (B, E), and *N. maritimus* (D, F). Putative EPS were detected as thread-like structures for *N. viennensis* and *N. maritimus* (D, F, white arrows), and granular structures for *N. franklandianus* and *N. maritimus* (E, F, white dashed arrows). *N. maritimus* cells collapsed due to SEM preparation were visible (F, gray arrow). Micrographs D-F are enlargements of areas marked with white dashed boxes in A–C, respectively.

For SEM of *N. viennensis*, *N. franklandianus,* and *N. maritimus* biofilms, the CG shown in Fig. 1 were prepared without removing the biofilm once stable nitrite production maxima were reached. SEM not only confirmed the multilayered and 3D nature of biofilms formed by *N. viennensis* and *N. franklandianus*, but also revealed the presence of putative EPS in the biofilms formed by both organisms (Fig. 3A, B, D, E). In *N. viennensis*, EPS formed thread-like structures that linked individual cells or clusters of cells together, whereas *N. franklandianus* exhibited granular EPS clusters between closely associated cells. *N. maritimus* biofilm structures were expectedly less extensive, but displayed a surprising amount of putative EPS (Fig. 3C and F) consisting of both an extensive granular EPS scaffold and minimal thread-like structures connecting few cells.

### Biofilms reveal a distinct transcriptional state in each species

**Figure 4:**
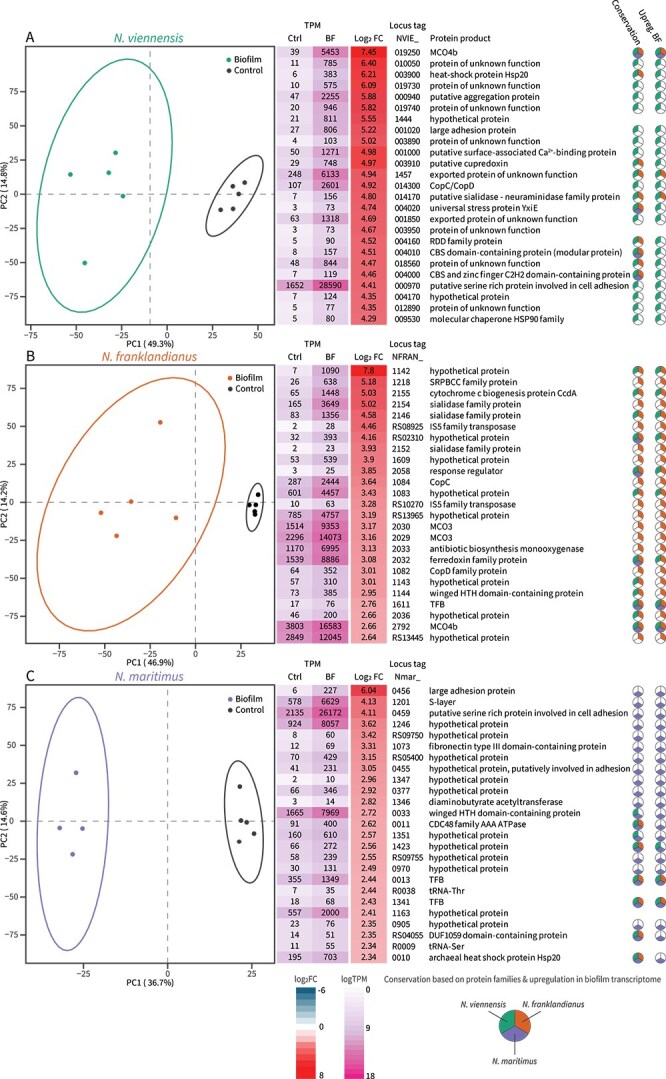
PCA plots and top 25 upregulated genes: PCA of log transformed expression data of *N*. *viennensis* (A), *N*. *franklandianus* (B), *N*. *maritimus* (C). Principal component 1 (PC1), explaining between 36.7%–49.3% of variance clearly separates conditions from each other, whereas principal component 2 (PC2), including 14.2%–14.8% of variance, describes the variance within conditions. Ellipses represent a confidence interval of 95%. PERMANOVA analysis indicated a significant difference between BF and Ctrl conditions based on the first two principal components (*N. viennensis*: *P* = 0.002; *N. franklandianus*: *P* = 0.008; *N. maritimus*: *P* = 0.009, cutoff = 0.01). Top 25 differentially expressed genes are ordered by log_2_ Fold Change (log_2_FC). Expression levels of genes, given as transcripts per million (TPM), are displayed for both planktonic controls (Ctrl) and biofilms (BF), and were color coded based on a log_2_ transformation scale (Datasets S2–S4). Wedged circles on the right indicate the genomic conservation of genes based on protein families and upregulation of genes in biofilms in *N. viennensis* (green), *N. franklandianus* (orange), or *N. maritimus* (purple).

To further characterize the differences of growth in biofilm to planktonic conditions, transcriptomic analyses were done for *N. viennensis*, *N. franklandianus,* and *N. maritimus* (Datasets S2–S4). Genes with a log_2_FC of >1.0/< −1.0 and an adjusted *P* value cut-off of 0.001 were considered significantly up- or downregulated. In *N*. *viennensis, N. franklandianus,* and *N. maritimus* 250/3187, 135/2768, 104/1972 genes were significantly upregulated and 59/3187, 20/2768, and 51/1972 genes were significantly downregulated, respectively. Principal component analysis (PCA) analyses revealed distinct transcriptomic states, clearly separating the different conditions on PC1 (Fig. 4). Variation between biological replicates of the same condition displayed on PC2 was more pronounced in biofilms than the planktonic controls, a pattern also observed in bacterial biofilms [34]. When comparing the protein families of the 25 strongest upregulated genes (Fig. 4), few responses were found to be conserved (see next section) among the transcriptomes of the three strains. Those included multicopper oxidases (MCOs) and the archaeal transcription initiation factor B (TFB). Most other highly upregulated genes within each strain were predominantly species-specific, with a slightly larger overlap among the soil strains (Fig. 4, “Upreg. BF”).

### Upregulation of few conserved protein families define biofilm formation across species

**Figure 5:**
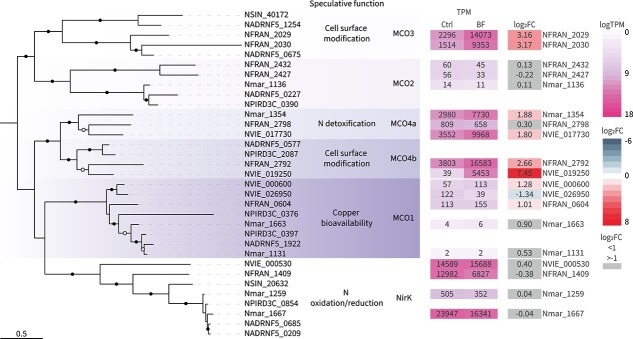
Phylogenetic tree of MCOs in *N. viennensis*, *N. franklandianus, N. sinensis*, *N. maritimus, N. adriaticus,* and *N. piranensis*. Significant and not significant log_2_FC are displayed and colored as gradient or gray respectively. TPM displayed for planktonic controls (ctrl) and biofilms (BF) were color coded based on a log_2_ transformation scale (Datasets S2–4). The scale bar in the bottom left corner indicates 50% sequence divergence.

The analysis was expanded to identify overlaps in upregulated protein families of all genes considered significantly upregulated. Protein families were created using amino acid sequences from selected AOA genomes, following previously used metrics (70% coverage, 35% identify cutoffs, see Materials and methods) [35]. These protein families were used to check for conservation (overlap) of genes between the three analyzed species. The majority of upregulated genes were linked to a protein family upregulated in only one out of each of the three species (195 in *N. viennensis*, 102 in *N. franklandianus,* and 83 in *N. maritimus*) (Fig. S2), emphasizing their species-specific responses. Out of all protein families, only three were significantly upregulated in biofilms of all three species (Fig. S2, Dataset S5). Of these, proteins of the multicopper oxidase family (MCO) were among the most differentially expressed in *N. viennensis* and *N. franklandianus*. A phylogenetic MCO tree expanding earlier work to include 143 AOA species was reconstructed and used to differentiate the upregulated MCOs potentially involved in biofilm formation (Fig. S3) [24]. A simplified version including only MCOs of the species investigated in this study was calculated (Fig. 5).

Although MCO1 and MCO2 genes were not highly transcribed in either planktonic conditions or biofilms, several MCO4 and MCO3 genes were found to be upregulated in biofilms. MCO4 genes can be further divided into two subtypes. The first subtype, MCO4a, was highly transcribed in planktonic conditions and further upregulated in biofilms of *N. viennensis* (NVIE_017730) and *N. maritimus* (Nmar_1354). In *N. franklandianus*, however, this subtype (NFRAN_2798) was only moderately transcribed in both conditions. The two soil strains harbor an additional subtype MCO4b, which is not present in *N. maritimus*. MCO4b was the most upregulated gene in *N. viennensis* (NVIE_019250) and also highly upregulated in *N. franklandianus* (NFRAN_2792). Additionally, *N. franklandianus* contains two copies (NFRAN_2029, NFRAN_2030) of type MCO3, which is not present in either *N. viennensis* or *N. maritimus*. Both MCO3s were among the most upregulated genes in biofilms of *N. franklandianus*.

The second protein family found upregulated in all three species was the archaeal tTFB, guiding the initiation of transcription in archaea [36]. At least one TFB gene was upregulated in biofilms of all three species. In *N. viennensis*, the highest expressed TFB (NVIE_012290) was further upregulated, whereas the expression of the other four TFBs remained unchanged (Dataset S6). In contrast, in *N. franklandianus*, no single TFB dominated, but three different TFBs (NFRAN_2995, NFRAN_3010, NFRAN_3143) were expressed equally under both conditions, whereas an upregulation of three other, less expressed TFBs (NFRAN_0924, NFRAN_1611, NFRAN_2944) was observed in biofilms. Only *N. maritimus* (which contains eight TFBs) clearly shifted its highest expressed TFB from Nmar_0517 in planktonic conditions to Nmar_0013 in biofilms (Dataset S6). A correlation analysis of the dominant biofilm TFB (Nmar_0013) in *N. maritimus* was performed (see Discussion) and found 37 gene correlations (30 positive, seven negative; adjusted *P* value ≤0.01, absolute value of cor ≥ 0.9).

The third protein family upregulated in all three species was a putative nitroreductase (Dataset S6).

The overlap of protein families of upregulated genes between the two soil strains was bigger than any other overlap and included sialidases, genes involved in urea transport or utilization, and several regulatory genes (Datasets S5, S6).

### Expression patterns and gene clusters of bona fide biofilm genes

**Figure 6:**
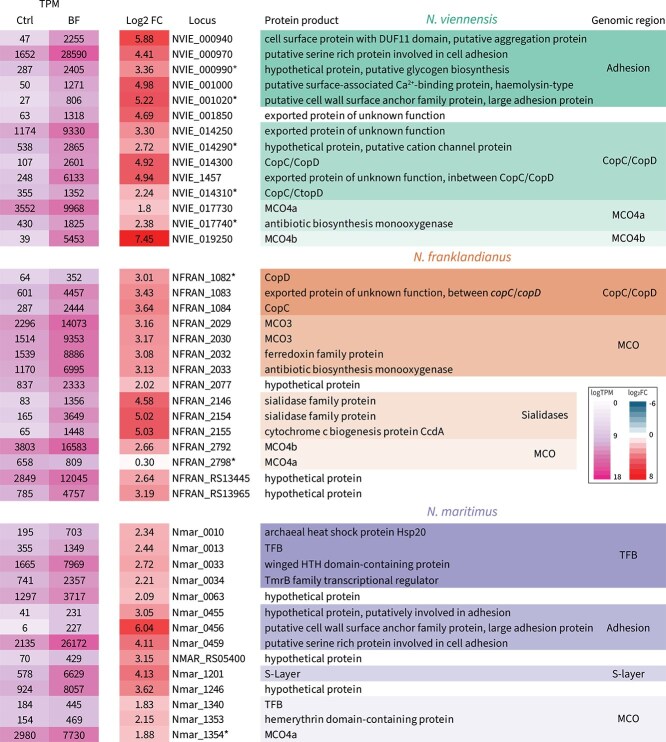
Bona fide biofilm genes. Top 50 significant log_2_FC biofilm genes of *N. viennensis*, *N. franklandianus, and N. maritimus* were cross-checked against the top 100 most highly expressed genes in biofilms (by TPM) to identify genes that are highly upregulated and highly expressed in AOA biofilms. The locus tags of manually added genes were marked with asterisks (^*^). These genes were either not included in the top 50 log_2_FC or had a TPM rank <100 biofilm genes, but were found in the direct vicinity of bona fide biofilm genes and followed the same general expression pattern. Shaded regions indicate genes with putative functional similarity and relative synteny (20 genes or less apart). Genes with “RS” indicate genes with a RefSeq annotation but no corresponding GenBank annotation. Selected gene annotations with hypothetical or unknown functions were manually curated when possible (Dataset S6). Either manually curated or automatic annotations are displayed.

Under the assumption that genes important under biofilm conditions should be upregulated and highly expressed in biofilms, a specific set of biofilm-associated genes was extracted (Fig. 6). In addition to selecting genes based on expression levels, we also considered their genomic organization to identify potential functional clusters, as genes with related functions are often co-localized. Firstly, the top 50 differentially upregulated genes for each species were cross-checked against the top 100 most highly expressed genes in biofilms (by TPM) to identify those that were both strongly upregulated and highly expressed in biofilms. Secondly, genes that were co-localized with the identified upregulated genes in the genome and followed the same expression pattern, but did not meet the chosen cutoffs, were also included (marked by an asterisk in Fig. 6).

Several genomic regions of biofilm associated genes, defined as continuous stretches of upregulated genes in the genome, were identified, especially in the soil strains. In contrast, biofilm-associated genes were more dispersed across the genome of *N. maritimus* (Fig. 6).

In *N. viennensis* and *N. franklandianus,* several MCOs were highly upregulated and highly expressed. Both MCO4a (NVIE_017730) in *N. viennensis* and MCO3 (NFRAN_2029–30) in *N. franklandianus* clustered with a gene annotated as antibiotic biosynthesis monooxygenase (NVIE_017740 and NFRAN_2033). In *N. franklandianus* a ferredoxin family protein gene (NFRAN_2032) was also part of this region. In N. *maritimus*, a hemerythrin (Nmar_1353) clustered with MCO4a (Nmar_1354) and was observed to be upregulated.

A genomic region involved in adhesion was found to be highly induced in the biofilms of the S-layer containing strains *N. viennensis* (NVIE_000940–001020) and *N. maritimus* (Nmar_0455–0459). This region included large adhesion proteins, ~1000 to 3000 amino acids in length, located next to other proteins potentially involved in adhesion, aggregation, and cell surface stability. Similar adhesion-related genes were not observed in *N. franklandianus*, which does not encode canonical S-layer genes [37].

In both soil strains, the *copCD*/*copC*-*copD* gene region (NVIE_014250–014310 and NFRAN_1082–1084) belonging to the copper resistance CopC and CopD protein families, was highly upregulated, with an additional putative cation channel protein (NVIE_014290) in direct vicinity in *N. viennensis*. Specific to *N. franklandianus*, a highly expressed region of sialidases was observed (NFRAN_2146 and NFRAN_2154).

TFBs were upregulated in all three species (Dataset S6). However, only in *N. maritimus* TFBs were highly upregulated and highly expressed (Fig. 6). Additionally, a secondary S-layer gene (Nmar_1201) was identified to be upregulated in *N. maritimus* biofilms.

### Genetic distribution of bona fide biofilm features in AOA

**Figure 7:**
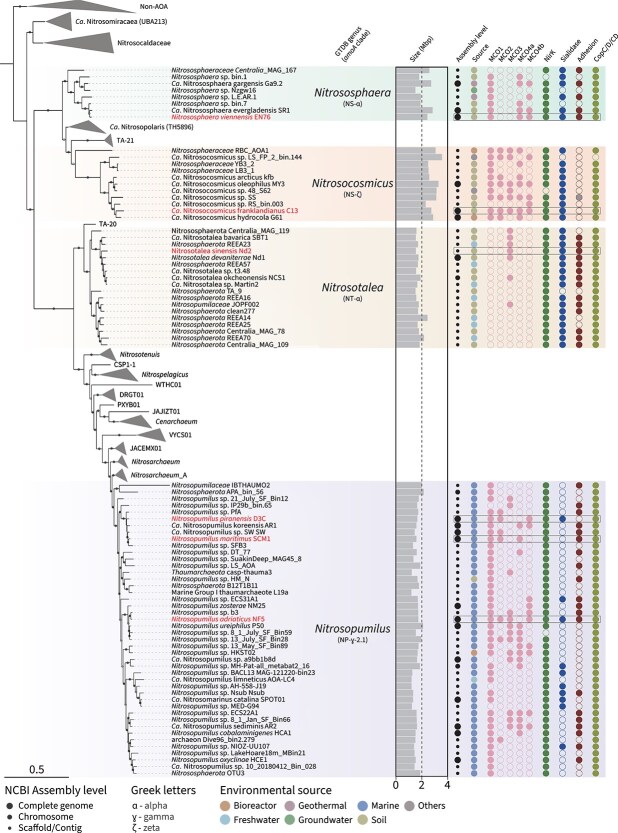
The distributions of genes of interest in genera *Nitrososphaera*, *Nitrosocosmicus*, *Nitrosotalea*, and *Nitrosopumilus*. The maximum likelihood phylogenomic tree of AOA was constructed based on a concatenation of 53 archaeal-specific markers (7589 columns, see Materials and Methods for details). Strains involved in this study are marked with red and boxed. Nodes with ultrafast bootstrap value ≥80% (60%) were indicated as solid (hollow) circles. The scale bar in the bottom left corner indicates 50% sequence divergence. The completeness and contamination of all shown genomes was ≥95% and < 5% respectively. Black circles represent different assembly levels according to NCBI. In brief, “complete genomes” are closed and contain no gaps, “chromosomes” are not closed and might still contain gaps whereas “scaffolds/contigs” are fragmented and are sure to contain gaps. A grey filled circle indicates an adhesion protein hit that is annotated as a PQQ dehydrogenase and not assumed to be an adhesion protein (Dataset S6). MCO, multicopper oxidase. An expanded version of this tree including additional information is provided in the supplementary materials (Fig. S4)**.**

To investigate the distribution of the identified bona fide biofilm genes (multicopper oxidases, sialidases, adhesion proteins, and *copCD*) among the diversity of AOA, a tree including 143 species was calculated and the presence of these genes in their genomes was analyzed (Fig. 7) (see Materials and Methods). MCOs exhibit considerable diversity, with clade specific patterns emerging. For instance, *Nitrososphaera* do not encode any MCO3, whereas *Nitrosotalea* only encode MCO3. Genes encoding for adhesion proteins are completely absent from the *Nitrosocosmicus* clade, although they are commonly found in other soil lineages and in some marine lineages. Sialidases are highly enriched in all soil strains but are not commonly found in the marine strains. Most AOA encode a *copC*/*copD* variant, underlining their dependence on copper.

## Discussion

In this study, we investigated the biofilm-forming capabilities of AOA from diverse habitats and identified common as well as taxon-specific expression patterns under these growth conditions. All tested AOA formed biofilms. Biofilm formation has been proposed as a stress response, or simply as a reaction to the presence of a suitable attachment surface [38], the latter aligning with the idea that biofilms represent the default growth mode in nature [16]. Given that these strains were cultivated under optimal planktonic conditions, the observed biofilm formation suggests that AOA may inherently tend toward a biofilm lifestyle. Soil strains, especially *N. franklandianus,* exhibited higher capacities for biofilm formation, as expected from previous studies on members of the *Nitrosocosmicus* showing a higher genomic potential for biofilm formation [20, 21, 27], or accelerated growth when surface associated [20]. Based on the presence of motility and chemotaxis genes, it was previously suggested that, although isolated from the same habitat, *N. piranensis* and *N. adriaticus* occupy different niches, floating in the water column or attached to marine snow particles, respectively [39]. Indeed, *N. adriaticus* showed significantly higher biofilm-forming capabilities than *N. piranensis* in our assay (Fig. 1), emphasizing that closely related species might occupy different microenvironments depending on their capacity for biofilm formation. The overall lower stability of biofilms from selected marine strains may be reflective of their environment, where communities attached to particles often undergo rapid successive changes [40]. Conversely, biofilms of terrestrial ecosystems might be more stable over prolonged periods of time.

### Attachment first

Two general strategies of biofilm formation were observed. *N. viennensis* and *N. maritimus* followed an attachment-first-strategy, gradually colonizing a surface, and subsequently building up a matrix. This strategy might be dependent on the presence of adhesion proteins in AOA that contain an S-layer. Adhesion proteins are highly expressed in biofilms of *N. viennensis* and *N. maritimus,* and are present in the genome of *N. adriaticus*, whereas *N. piranensis* lacks these genes, potentially explaining its reduced biofilm-forming capabilities. Adhesion proteins are distributed irregularly among the different genomes of AOA hinting towards niche specialization by different AOA (Fig. 7). A genetic survey linked the absence of adhesion proteins to the S-layer free genus *Nitrosocosmicus* [41]. The most probable location of the adhesion proteins is therefore within the S-Layer. However, the possibility that these proteins are incorporated into the EPS cannot be ruled out. Several other proteins are potentially involved in adhesion. For example, NVIE_000970 encodes a DUF11 domain that was also found encoded in a protein involved in cell surface stabilization during the aggregation of *Methanothermobacter* sp., CaT2 [42]. Additionally, a putative Ca^2+^-binding protein (NVIE_001010) is present in the adhesion region. Calcium, known to enhance adhesion in *Staphylococcus aureus* and *Staphylococcus epidermidis* [43], could similarly play a structural role in the biofilms of *N. viennensis*. In this context, a highly upregulated putative cation channel protein (NVIE_014290) may modulate Ca^2+^ levels of the biofilm. Adhesion proteins are recognized as key components of environmental biofilms [44], and our findings suggest that these proteins are likely crucial for enabling the attachment strategy observed in these particular species.

### Aggregation first

*N. franklandianus* exhibited an aggregation-first-strategy similar to oral bacteria, typically aggregating in clusters of mixed cell types [45]. Cells deposited on the CG as preformed suspended aggregates and 3D structures were formed rapidly. However, it is unclear how aggregates attach to surfaces, as specific mechanisms for attachment, such as homologs of the aforementioned adhesion proteins, are not present in the genomes (Fig. 7). Cell surface modifications and EPS production might not only enable aggregation of cells, but also attachment to surfaces. Based on our observations of strong biofilm formation by *N. franklandianus* under otherwise optimal planktonic conditions, along with previous observations of *Nitrosocosmicus* spp. primarily occurring in suspended aggregates or as part of biofilms [20, 21, 23, 27, 46], we suggest that members of this clade are well-adapted to a biofilm lifestyle in soil environments.

### MCOs in biofilm formation

MCOs are commonly found in AOA genomes and show active transcription across various conditions [24, 47, 48], although the biological function of most of them remains unknown. MCOs couple the oxidation of a substrate, that can be either organic or metal ions, to the reduction of oxygen to water [49], but can have a wide range of substrates, sometimes even acting promiscuously [50]. MCOs have previously been speculated to be involved in the second step of ammonia oxidation as a functional homologue to bacterial hydroxylamine dehydrogenase (also known as hydroxylamine oxidoreductase, HAO) [47, 51], but the fact that MCOs are not conserved in all AOA genomes suggests that they are not involved in a central pathway [24]. The patchy distribution of different MCOs among AOA instead points towards diverse functions.

In *N. viennensis*, MCO1, 4a, and 4b were upregulated in biofilms (Fig. 5) and were previously found to be also highly upregulated under copper limitation, potentially oxidizing Cu^+^ to Cu^2+^ for bioavailability [48], a known function for MCOs [52]. Contrastingly, in copper limited *N. maritimus,* the only upregulated MCO was of type MCO1 [53]. Combining these observations, it is likely that MCO1 is indeed used for copper acquisition. In further support of this hypothesis, an upregulation of MCO1 is found in both soil strains along with CopC/D transporters. This may be necessary because the EPS matrix is known to sequester positively charged ions like copper [54], trapping them and thus requiring increased activity of the uptake machinery. This was further supported by the presence of a cation channel protein located adjacent to *copC*/*copD*, which could supply other cations, such as Ca^2+^, that are bound by the biofilm matrix. It is plausible that copper acquisition genes in *N. maritimus* (CopD and MCO1) were not found to be upregulated due to the single-layer architecture of the marine biofilm not impairing availability of copper.

MCO4a was moderately to highly expressed in planktonic cultures and biofilms of all three species, and additionally highly upregulated in *N. viennensis* and *N. maritimus* biofilms (Fig. 6). Recent functional evidence for MCO4a from *N. maritimus* has shown the capability to produce HNO from NH_2_OH which was proposed as a waste production pathway, presumably to prevent the accumulation of NH_2_OH to cytotoxic levels [55]. While the downstream processing of HNO remains unknown, the upregulation of MCO4a still points to elevated nitrosative stress, likely due to both NH_2_OH and HNO in biofilms, where the close proximity of cells could lead to higher local concentrations of reactive molecules.

The function of the upregulated nitroreductase domain containing protein in all strains is currently unknown. However, a gene with a similar domain was also observed to be upregulated in biofilms of *Thermotoga maritima* along with genes relieving oxidative stress [56]. In general, nitroreductases could serve to handle toxic by-products with nitro groups [57] and have been observed to cause a release of nitric oxide [58] and may therefore help to balance the intercellular redox state of the cell.

Although MCO4a and MCO4b are overall similar, an alignment revealed that the T1 copper center in MCO4a is coordinated by histidines and a leucine, whereas the MCO4b copper center is coordinated by histidines and methionine [48] (found in supplementary material of cited paper). The methionine’s thioether group has been shown to modulate the redox potential of the coordinated Cu(II) [59] and might therefore be enabling a different function. The functional classification of MCOs using only primary structure is, however, significantly limited [50].

MCO4b and MCO3 were the most highly upregulated MCOs in biofilms of *N. viennensis* and *N. franklandianus* respectively. It is possible that these MCOs modify cell surfaces by oxidizing components like glycoproteins and in particular the glycosylated cell envelope. That MCO4b was the highest upregulated gene under both copper limitation and in biofilms of *N. viennensis* suggests a dual role of these cell surface modifications: (i) modifying cell surface structures to enable biofilm formation, and/or (ii) aiding in the sequestration of positively charged ions, such as copper [60]. Additionally, MCOs in *N. franklandianus* were also highly expressed in planktonic conditions (Fig. 5.), which is in line with the observed continuous formation of cell aggregates in this strain.

Several bona fide biofilm genes are also in close association with MCOs. The soil strains encode an “antibiotic biosynthesis monooxygenase” next to MCO4a and MCO3, respectively, indicating another oxygenase function that could modify the cell coat (rather than being involved in antibiotic biosynthesis). A putative electron carrier, the ferredoxin/flavodoxin family protein, is directly adjacent to an MCO in all three strains, potentially indicating the importance of supplying electrons to MCOs (Fig. 6, Datasets S2–S4).

### Transcription factor B as regulators in AOA biofilms

Transcription factors TBP and TFB, homologs of the eukaryotic basal transcription factors, are consistently found in all archaea and are essential for transcription. Similar to *Halobacterium* sp. NRC-1, AOA encode several TFB proteins (but only one TBP, differing from Halobacteria) in their genomes that could be involved in global gene regulation [61]. An up-regulation of at least one TFB in the biofilm phenotype was found in each species with distinct patterns: an increase in the dominant TFB (*N. viennensis*), an increase in three lowly expressed TFBs with the dominant TFBs remaining unchanged (*N. franklandianus*), and an increase in three lowly expressed TFBs, one of which becomes the dominant biofilm TFB (*N. maritimus*). Even though the dominant TFBs do not change in soil strains, the switch of dominant TFBs in *N. maritimus* may suggest a more drastic phenotype change in this organism. Indeed 30 genes were positively correlated with the upregulation of the TFB protein dominating in biofilm including all but one (Nmar_0010) of the bona fide biofilm genes of *N. maritimus* (Fig. 6).

### Biofilm features specific to soil strains

Prominent features of biofilms in soil AOA include an increased BAR and a more complex three dimensional architecture. These features suggest a dynamic system where resources may be fluctuating. This is a likely reason for the previously discussed upregulation of copper acquisition genes, and may also account for the upregulation of urease and urea transport genes in *N. viennensis* and *N. franklandianus* (Dataset S5*)* in the absence of urea. These could allow for the rapid utilization of urea or other nitrogenous compounds when present. Cells in biofilms have been shown to employ highly efficient strategies for capturing diverse nutrients, surpassing that of free-living bacterial cells [62].

A set of genes putatively involved in biofilm formation, including CAZymes, was previously proposed for *N. viennensis* and *N. franklandianus* [20, 24]. Except for genes involved in adhesion, those genes were not differentially expressed in our datasets (Datasets S6). This might be in line with reports of polysaccharides sometimes being only a minor component in the EPS of environmental biofilms, highlighting the importance of other factors like adhesion proteins [63]. Regardless of the composition, putative EPS were identified via SEM for all imaged species. Although dehydration during SEM sample preparation can lead to EPS collapse, often leading to filamentous structures [64] as seen for mostly *N. viennensis* (Fig. 3D), the amount and architecture of putative EPS structures differed strongly between *N. viennensis, N. franklandianus*, and *N. maritimus* (Fig. 3), pointing towards different EPS compositions of AOA from diverse habitats. For example, the recent differences observed in N-glycosylation patterns between *N. viennensis* and *N. piranensis* [65].

Sialidase encoding genes were upregulated in both soil strains, but prominently only in *N. franklandianus*. They have been shown to modify glycoproteins and glycolipids by cleaving sialic acid, which are common compounds of environmental biofilms [66, 67]. Furthermore, sialidase genes are present in the genomes of most AOA from soil and generally absent in the genomes of marine AOA (Fig. 7). Adjacent to the two sialidase genes in *N. franklandianus* is a gene for an electron carrier protein (ccd biogenesis protein, NFRAN_2155), which could be essential for supplying electrons to the sialidases, ensuring their activity and highlighting the importance of building up an EPS matrix.

### Specific responses in marine strain

Similar to *N. viennensis,* the marine strain *N. maritimus* relies on adhesion proteins for initial attachment. Instead of MCOs for surface modification, we observed a strong upregulation of an alternative S-layer protein that is likely important for biofilm formation and could possibly be needed to allow the introduction of the adhesion protein into the S-Layer.

Hemerythrin was found to be part of *N. maritimus* bona fide biofilm genes. Microbial communities associated with marine snow undergo complex successional changes [68]. While sinking, oxygen concentration decreases and hemerythrin could therefore act as an oxygen carrier [69], similar to the situation in *Methylococcus capsulatus,* where bacteriohemerythrin protein has been shown to be highly upregulated under low oxygen conditions [70]. The NP-gamma clade did not exhibit a clear pattern with regards to the bona fide biofilm genes identified in this paper (Fig. 7). Nevertheless, strains from marine sediments (i.e. *Nitrosopumilus ureiphilus* PS0 [71] and *Nitrosopumilus sediminis* AR2 [72]) were found to contain identified homologs of potential biofilm genes (MCO3 and MCO3/adhesion respectively). While not conclusive, this would suggest that they may employ similar biofilm strategies as observed here. Putative adhesion proteins were also found in genomes from deep sea sediments (i.e. NP-theta, NP-eta, Fig. S4), but not all (i.e. NP-delta representative, Fig. S4) [73].

## Conclusion

**Figure 8:**
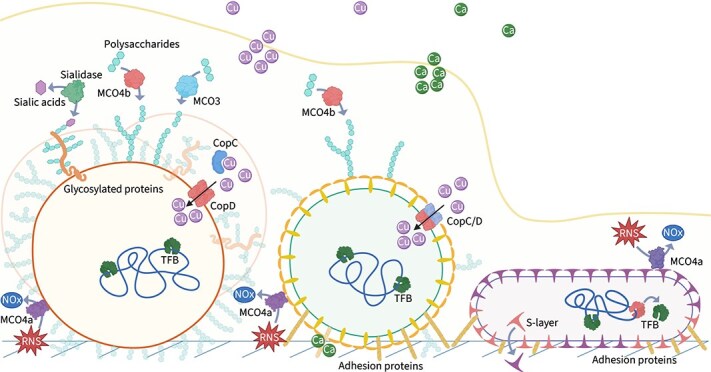
Schematic overview of AOA biofilms highlighting a subset of functional genes and their putative functions. Represented genes were chosen based on the common themes emerging from the bona fide biofilm gene analysis shown in Fig. 6: Adhesion, strong upregulation and expression of MCOs (potential cell surface modification), copper acquisition machinery, and transcriptional regulation. Created in BioRender. Dreer, M. (2025) https://BioRender.com/d7kir2e.

We have demonstrated that all tested AOA have the capacity to form biofilms, with soil strains showing the highest propensity for biofilm formation. Transcriptome analysis of biofilm cultures suggests a reliance on cell surface modifications and/or adhesion, for which two strategies were observed (Fig. 8). Distinct expression patterns illustrate that mostly individual solutions have evolved for the shared growth mode of biofilm formation in AOA, probably driven by the different ecological niches.

AOA are among the few archaea globally distributed with great ecological success. This is likely in part due to their high energy efficiency, encoding the most efficient CO_2_ fixation pathway [74], and their high affinities for ammonia [15, 75]. Their broad ecological success could, however, also be attributed to their ability to form biofilms. From this work, it is clear that to understand the role of AOA in the environment, they must also be studied in the context of biofilms. This is especially true for AOA derived from soils and may even be imperative for the genus *Nitrosocosmicus*. Improvements in biofilm cultivation of AOA analogous to those achieved for planktonic cultures (i.e. bioreactors) [29, 76] would greatly benefit this endeavor and are promising as biofilms of AOA have been observed to form in scaled up systems [29]. This would allow for more extensive biofilm characterization (i.e. EPS). The study presented here sets the stage for future investigations of the biofilm structures as well as the activities and ecophysiological effects of AOA in biofilms.

## Supplementary Material

Dataset_S1_growth_data_wraf182

Dataset_S2_Nviennensis_wraf182

Dataset_S3_Nfranklandianus_wraf182

Dataset_S4_Nmaritimus_wraf182

Dataset_S5_conserved_upreg_protein_families_Venn_diagramm_wraf182

Dataset_S6_figures_discussion_data_wraf182

Fig_S5_wraf182

Supplementary_clean_FINAL_wraf182

## Data Availability

The whole-transcriptome data generated in this study have been deposited in the NCBI BioProject database under the accession project number (PRJNA1156597), Submission ID: SUB14703162 (https://www.ncbi.nlm.nih.gov/sra/PRJNA1156597). All additional datasets generated during the current study are provided in the associated datasets or is available in the Pribasnig repository, under following link: https://github.com/pribasnig/Biofilm_NV_NF_NM.

## References

[1] Könneke M, Bernhard AE, de La Torre JR. et al. Isolation of an autotrophic ammonia-oxidizing marine archaeon. *Nature* 2005;437:543–6. 16177789 10.1038/nature03911

[2] Treusch AH, Leininger S, Kietzin A. et al. Novel genes for nitrite reductase and Amo-related proteins indicate a role of uncultivated mesophilic crenarchaeota in nitrogen cycling. *Environ Microbiol* 2005;7:1985–95. 10.1111/j.1462-2920.2005.00906.x 16309395

[3] Winogradsky S . Recherches Sur les organismes de la nitrification. *Ann Inst Pateur (Paris)* 1890;4:213–31.

[4] Daims H, Lebedeva EV, Pjevac P. et al. Complete nitrification by *Nitrospira* bacteria. *Nature* 2015;528:504–9. 10.1038/nature16461 26610024 PMC5152751

[5] van Kessel MAHJ, Speth DR, Albertsen M. et al. Complete nitrification by a single microorganism. *Nature* 2015;528:555–9. 10.1038/nature16459 26610025 PMC4878690

[6] Freing A, Wallace DWR, Bange HW. Global oceanic production of nitrous oxide. *Philos Trans R Soc B, Biol Sci* 2012;367:1245–55. 10.1098/rstb.2011.0360 PMC3306629 22451110

[7] Santoro AE, Buchwald C, McIlvin MR. et al. Isotopic signature of N_2_O produced by marine ammonia-oxidizing archaea. *Science* 2011;333:1282–5. 10.1126/science.1208239 21798895

[8] Yao H, Gao Y, Nicol GW. et al. Links between ammonia oxidizer community structure, abundance, and nitrification potential in acidic soils. *Appl Environ Microbiol* 2011;77:4618–25. 10.1128/AEM.00136-11 21571885 PMC3127715

[9] Norton J, Ouyang Y. Controls and adaptive management of nitrification in agricultural soils. *Front Microbiol* 2019;10:01931. 10.3389/fmicb.2019.01931 PMC6728921 31543867

[10] Nunoura T, Takaki Y, Hirai M. et al. Hadal biosphere: insight into the microbial ecosystem in the deepest ocean on earth. *Proc Natl Acad Sci USA* 2015;112:E1230–6. 10.1073/pnas.1421816112 25713387 PMC4371994

[11] Zhang L-M, Wang M, Prosser JI. et al. Altitude ammonia-oxidizing bacteria and archaea in soils of Mount Everest. *FEMS Microbiol Ecol* 2009;70:208–17. 10.1111/j.1574-6941.2009.00775.x 19780828

[12] Leininger S, Urich T, Schloter M. et al. Archaea predominate among ammonia-oxidizing prokaryotes in soils. *Nature* 2006;442:806–9. 10.1038/nature04983 16915287

[13] Karner MB, Delong EF, Karl DM. Archaeal dominance in the mesopelagic zone of the Pacific Ocean. *Nature* 2001;409:507–10. 10.1038/35054051 11206545

[14] Di HJ, Cameron KC, Shen J-P. et al. Ammonia-oxidizing bacteria and archaea grow under contrasting soil nitrogen conditions. *FEMS Microbiol Ecol* 2010;72:386–94. 10.1111/j.1574-6941.2010.00861.x 20370827

[15] Martens-Habbena W, Berube PM, Urakawa H. et al. Ammonia oxidation kinetics determine niche separation of nitrifying archaea and bacteria. *Nature* 2009;461:976–9. 10.1038/nature08465 19794413

[16] Flemming H-C, Wuertz S. Bacteria and archaea on earth and their abundance in biofilms. *Nat Rev Microbiol* 2019;17:247–60. 10.1038/s41579-019-0158-9 30760902

[17] Lloyd CC, Brown S, Balmonte JP. et al. Particles act as ‘specialty centers’ with expanded enzymatic function throughout the water column in the western North Atlantic. *Front Microbiol* 2022;13:882333. 10.3389/fmicb.2022.882333 36246226 PMC9553992

[18] Tourna M, Stieglmeier M, Spang A. et al. *Nitrososphaera viennensis*, an ammonia oxidizing archaeon from soil. *Proc Natl Acad Sci USA* 2011;108:8420–5. 10.1073/pnas.1013488108 21525411 PMC3100973

[19] Rinke C, Chuvochina M, Mussig AJ. et al. A standardized archaeal taxonomy for the genome taxonomy database. *Nat Microbiol* 2021;6:946–59. 10.1038/s41564-021-00918-8 34155373

[20] Jung MY, Kim JG, Sinninghe Damsté JS. et al. A hydrophobic ammonia-oxidizing archaeon of the *Nitrosocosmicus* clade isolated from coal tar-contaminated sediment. *Environ Microbiol Rep* 2016;8:983–92. 10.1111/1758-2229.12477 27700018

[21] Liu L, Li S, Han J. et al. A two-step strategy for the rapid enrichment of *Nitrosocosmicus*-like ammonia-oxidizing Thaumarchaea. *Front Microbiol* 2019;10:00875. 10.3389/fmicb.2019.00875 PMC6491936 31105671

[22] Klein T, Poghosyan L, Barclay JE. et al. Cultivation of ammonia-oxidising archaea on solid medium. *FEMS Microbiol Lett* 2022;369:fnac029. 10.1093/femsle/fnac029 35323924 PMC9072212

[23] Lehtovirta-Morley LE, Ross J, Hink L. et al. Isolation of ‘*Candidatus* Nitrosocosmicus franklandus’, a novel ureolytic soil archaeal ammonia oxidiser with tolerance to high ammonia concentration. *FEMS Microbiol Ecol* 2016;92:fiw057. 10.1093/femsec/fiw057 26976843 PMC4830249

[24] Kerou M, Offre P, Valledor L. et al. Proteomics and comparative genomics of *Nitrososphaera viennensis* reveal the core genome and adaptations of archaeal ammonia oxidizers. *Proc Natl Acad Sci USA* 2016;113:E7937–46. 27864514 10.1073/pnas.1601212113 PMC5150414

[25] Daebeler A, Herbold CW, Vierheilig J. et al. Cultivation and genomic analysis of “*Candidatus* Nitrosocaldus islandicus,” an obligately thermophilic, ammonia-oxidizing Thaumarchaeon from a hot spring biofilm in Graendalur Valley, Iceland. *Iceland Front Microbiol* 2018;9:00193. 10.3389/fmicb.2018.00193 PMC5817080 29491853

[26] Slosser T, Wenick M, Markert E. et al. Novel hot spring Thermoproteota support vertical inheritance of ammonia oxidation and carbon fixation in *Nitrososphaeria*. *Access Microbiol* 2025;7:000931.v4. 10.1099/acmi.0.000931.v4 PMC12041475 40309222

[27] Sauder LA, Albertsen M, Engel K. et al. Cultivation and characterization of *Candidatus* Nitrosocosmicus exaquare, an ammonia-oxidizing archaeon from a municipal wastewater treatment system. *ISME J* 2017;11:1142–57. 10.1038/ismej.2016.192 28195581 PMC5398378

[28] Yin Z, Bi X, Xu C. Ammonia-oxidizing archaea (AOA) play with ammonia-oxidizing bacteria (AOB) in nitrogen removal from wastewater. *Archaea 2018* 2018;2018:1–9. 10.1155/2018/8429145 PMC6158934 30302054

[29] Melcher M, Hodgskiss LH, Mardini MA. et al. Analysis of biomass productivity and physiology of *Nitrososphaera viennensis* grown in continuous culture. *Front Microbiol* 2023;14:1076342. 10.3389/fmicb.2023.1076342 36876066 PMC9978112

[30] Lehtovirta-Morley LE, Ge C, Ross J. et al. Characterisation of terrestrial acidophilic archaeal ammonia oxidisers and their inhibition and stimulation by organic compounds. *FEMS Microbiol Ecol* 2014;89:542–52. 10.1111/1574-6941.12353 24909965 PMC4261999

[31] Bayer B, Vojvoda J, Reinthaler T. et al. *Nitrosopumilus adriaticus* sp. nov. and *Nitrosopumilus piranensis* sp. nov., two ammonia-oxidizing archaea from the Adriatic Sea and members of the class *Nitrososphaeria*. *Int J Syst Evol Microbiol* 2019;69:1892–902. 10.1099/ijsem.0.003360 30938665 10.1099/ijsem.0.003360

[32] Vert M, Doi Y, Hellwich K-H. et al. Terminology for biorelated polymers and applications (IUPAC recommendations 2012). *Pure Appl Chem* 2012;84:377–410. 10.1351/PAC-REC-10-12-04

[33] Hink L, Bachtsevani E, Meng Y. et al. Acidotolerant soil nitrite oxidiser ‘*Candidatus* Nitrobacter laanbroekii’ NHB1 alleviates constraints on growth of acidophilic soil ammonia oxidisers. 2024;2024: bioRxiv 2024.07.06.601931. 10.1101/2024.07.06.601931 PMC12815253 41561309

[34] Stewart PS, Franklin MJ. Physiological heterogeneity in biofilms. *Nat Rev Microbiol* 2008;6:199–210. 10.1038/nrmicro1838 18264116

[35] Abby SS, Kerou M, Schleper C. Ancestral reconstructions decipher major adaptations of ammonia-oxidizing archaea upon radiation into moderate terrestrial and marine environments. *mBio* 2020;11:e02371–20. 33051370 10.1128/mBio.02371-20 PMC7554672

[36] Reeve JN . Archaeal chromatin and transcription. *Mol Microbiol* 2003;48:587–98. 10.1046/j.1365-2958.2003.03439.x 12694606

[37] Nicol GW, Hink L, Gubry-Rangin C. et al. Genome sequence of “*Candidatus* Nitrosocosmicus franklandus” C13, a terrestrial ammonia-oxidizing archaeon. *Microbiol Resour Announc* 2019;8:e00435–19. 31582432 10.1128/MRA.00435-19 PMC6776761

[38] Jefferson KK . What drives bacteria to produce a biofilm? *FEMS Microbiol Lett* 2004;236:163–73. 10.1111/j.1574-6968.2004.tb09643.x 15251193

[39] Bayer B, Vojvoda J, Offre P. et al. Physiological and genomic characterization of two novel marine thaumarchaeal strains indicates niche differentiation. *ISME J* 2015;10:1051–63. 26528837 10.1038/ismej.2015.200 PMC4839502

[40] Datta MS, Sliwerska E, Gore J. et al. Microbial interactions lead to rapid micro-scale successions on model marine particles. *Nat Commun* 2016;7:11965. 10.1038/ncomms11965 27311813 PMC4915023

[41] Han S, Kim S, Sedlacek CJ. et al. Adaptive traits of *Nitrosocosmicus* clade ammonia-oxidizing archaea. *mBio* 2024;15:e02169–24. 10.1128/mbio.02169-24 PMC11559005 39360821

[42] Sumikawa K, Kosaka T, Mayahara N. et al. An aggregation-defective mutant of *Methanothermobacter* sp. CaT2 reveals unique protein-dependent aggregation. *Microbes Environ* 2019;34:244–51. 10.1264/jsme2.ME19014 31189768 PMC6759345

[43] Thomas VL, Sanford BA, Ramsay MA. Calcium- and mucin-binding proteins of staphylococci. *J Gen Microbiol* 1993;139:623–9. 10.1099/00221287-139-3-623 8473868

[44] Larsen P, Nielsen JL, Dueholm MS. et al. Amyloid adhesins are abundant in natural biofilms. *Environ Microbiol* 2007;9:3077–90. 10.1111/j.1462-2920.2007.01418.x 17991035

[45] Kolenbrander PE . Intergeneric coaggregation among human oral bacteria and ecology of dental plaque. *Ann Rev Microbiol* 1988;42:627–56. 10.1146/annurev.mi.42.100188.003211 3060002

[46] Alves RJE, Kerou M, Zappe A. et al. Ammonia oxidation by the arctic terrestrial thaumarchaeote *Candidatus* Nitrosocosmicus arcticus is stimulated by increasing temperatures. *Front Microbiol* 2019;10:1571. 10.3389/fmicb.2019.01571 31379764 PMC6657660

[47] Walker CB, de La Torre JR, Klotz MG. et al. *Nitrosopumilus maritimus* genome reveals unique mechanisms for nitrification and autotrophy in globally distributed marine crenarchaea. *Proc Natl Acad Sci USA* 2010;107:8818–23. 10.1073/pnas.0913533107 20421470 PMC2889351

[48] Reyes C, Hodgskiss LH, Kerou M. et al. Genome wide transcriptomic analysis of the soil ammonia oxidizing archaeon *Nitrososphaera viennensis* upon exposure to copper limitation. *ISME J* 2020;14:2659–74. 10.1038/s41396-020-0715-2 32665710 PMC7785015

[49] Solomon EI, Sundaram UM, Machonkin TE. Multicopper oxidases and oxygenases. *Chem Rev* 1996;96:2563–606. 11848837 10.1021/cr950046o

[50] Martins LO, Durão P, Brissos V. et al. Laccases of prokaryotic origin: enzymes at the interface of protein science and protein technology. *Cell Mol Life Sci* 2015;72:911–22. 10.1007/s00018-014-1822-x 25572294 PMC11113980

[51] Stahl DA, de La Torre JR. Physiology and diversity of ammonia-oxidizing archaea. *Ann Rev Microbiol* 2012;66:83–101. 10.1146/annurev-micro-092611-150128 22994489

[52] Singh SK, Grass G, Rensing C. et al. Cuprous oxidase activity of CueO from *Escherichia coli*. *J Bacteriol* 2004;186:7815–7. 10.1128/JB.186.22.7815-7817.2004 15516598 PMC524913

[53] Qin W, Amin SA, Lundeen RA. et al. Stress response of a marine ammonia-oxidizing archaeon informs physiological status of environmental populations. *ISME J* 2017;12:508–19. 10.1038/ismej.2017.186 29053148 PMC5776466

[54] Fathollahi A, Coupe SJ, El-Sheikh AH. et al. Cu(II) biosorption by living biofilms: isothermal, chemical, physical and biological evaluation. *J Environ Manag* 2021;282:111950. 10.1016/j.jenvman.2021.111950 33465714

[55] Voland RW, Wang H, Abruña HD. et al. Nitrous oxide production via enzymatic nitroxyl from the nitrifying archaeon *Nitrosopumilus maritimus*. *Proc Natl Acad Sci USA* 2025;122:e2416971122. 10.1073/pnas.2416971122 39823305 PMC11761707

[56] Pysz MA, Conners SB, Montero CI. et al. Transcriptional analysis of biofilm formation processes in the anaerobic, hyperthermophilic bacterium *Thermotoga maritima*. *Environ Microbiol* 2004;70:6098–112. 10.1128/AEM.70.10.6098-6112.2004 PMC522082 15466556

[57] Roldán MD, Pérez-Reinado E, Castillo F. et al. Reduction of polynitroaromatic compounds: the bacterial nitroreductases. *FEMS Microbiol Rev* 2008;32:474–500. 10.1111/j.1574-6976.2008.00107.x 18355273

[58] Hibbard HAJ, Reynolds MM. Synthesis of novel nitroreductase enzyme-activated nitric oxide prodrugs to site-specifically kill bacteria. *Bioorg Chem* 2019;93:103318. 10.1016/j.bioorg.2019.103318 31586703

[59] Xu F, Palmer AE, Yaver DS. et al. Targeted mutations in a *Trametes villosa* laccase. Axial perturbations of the T1 copper. *J Biol Chem* 1999;274:12372–5. 10.1074/jbc.274.18.12372 10212209

[60] Varki A., Cummings R.D., Esko J.D.. et al. (eds.). Essentials of Glycobiology, 4th edn. Cold Spring Harbor (NY): Cold Spring Harbor Laboratory Press, 2022. 20301239

[61] Coker JA, DasSarma S. Genetic and transcriptomic analysis of transcription factor genes in the model halophilic archaeon: coordinate action of TbpD and TfbA. *BMC Genet* 2007;8:61. 10.1186/1471-2156-8-61 17892563 PMC2121645

[62] Flemming H-C, Wingender J, Szewzyk U. et al. Biofilms: an emergent form of bacterial life. *Nat Rev Microbiol* 2016;14:563–75. 10.1038/nrmicro.2016.94 27510863

[63] Frølund B, Palmgren R, Keiding K. et al. Extraction of extracellular polymers from activated sludge using a cation exchange resin. *Water Res* 1996;30:1749–58. 10.1016/0043-1354(95)00323-1

[64] Dohnalkova AC, Marshall MJ, Arey BW. et al. Imaging hydrated microbial extracellular polymers: comparative analysis by electron microscopy. *Appl Environ Microbiol* 2011;77:1254–62. 10.1128/AEM.02001-10 21169451 PMC3067245

[65] Nakagawa S, Yagi H, Suyama T. et al. Exploring protein N-glycosylation in ammonia-oxidizing *Nitrososphaerota* archaea through glycoproteomic analysis. *mBio* 2025;16:e03859–24. 40387319 10.1128/mbio.03859-24 PMC12153357

[66] Pinel ISM, Kleikamp HBC, Pabst M. et al. Sialic acids: an important family of carbohydrates overlooked in environmental biofilms. *Appl Sci* 2020;10:7694. 10.3390/app10217694

[67] Soong G, Muir A, Gomez MI. et al. Bacterial neuraminidase facilitates mucosal infection by participating in biofilm production. *J Clin Invest* 2006;116:2297–305. 10.1172/JCI27920 16862214 PMC1513050

[68] Alldredge AL, Silver MW. Characteristics, dynamics and significance of marine snow. *Prog Oceanogr* 1988;20:41–82. 10.1016/0079-6611(88)90053-5

[69] Alvarez-Carreño C, Becerra A, Lazcano A. Molecular evolution of the oxygen-binding hemerythrin domain. *PLoS One* 2016;11:e0157904. 27336621 10.1371/journal.pone.0157904 PMC4919030

[70] Kalyuzhnaya MG, Yang S, Rozova ON. et al. Highly efficient methane biocatalysis revealed in a methanotrophic bacterium. *Nat Commun* 2013;4:2785. 10.1038/ncomms3785 24302011

[71] Qin W, Heal KR, Ramdasi R. et al. *Nitrosopumilus maritimus* gen. Nov., sp. nov., *Nitrosopumilus cobalaminigenes* sp. nov., *Nitrosopumilus oxyclinae* sp. nov., and *Nitrosopumilus ureiphilus* sp. nov., four marine ammonia-oxidizing archaea of the phylum *Thaumarchaeota*. *Int J Syst Evol Microbiol* 2017;67:5067–79. 29034851 10.1099/ijsem.0.002416

[72] Park B-J, Park S-J, Yoon D-N. et al. Cultivation of autotrophic ammonia-oxidizing archaea from marine sediments in coculture with sulfur-oxidizing bacteria. *Appl Environ Microbiol* 2010;76:7575–87. 10.1128/AEM.01478-10 20870784 PMC2976178

[73] Kerou M, Ponce-Toledo RI, Zhao R. et al. Genomes of Thaumarchaeota from deep sea sediments reveal specific adaptations of three independently evolved lineages. *ISME J* 2021;15:2792–808. 10.1038/s41396-021-00962-6 33795828 PMC8397731

[74] Könneke M, Schubert DM, Brown PC. et al. Ammonia-oxidizing archaea use the most energy-efficient aerobic pathway for CO_2_ fixation. *Proc Natl Acad Sci USA* 2014;111:8239–44. 10.1073/pnas.1402028111 24843170 PMC4050595

[75] Jung MY, Sedlacek CJ, Kits KD. et al. Ammonia-oxidizing archaea possess a wide range of cellular ammonia affinities. *ISME J* 2022;16:272–83. 10.1038/s41396-021-01064-z 34316016 PMC8692354

[76] Hurley SJ, Elling FJ, Könneke M. et al. Influence of ammonia oxidation rate on thaumarchaeal lipid composition and the TEX_86_ temperature proxy. *Proc Natl Acad Sci USA* 2016;113:7762–7. 10.1073/pnas.1518534113 27357675 PMC4948339

