# Supplementary figures and images for: Biofilm lifestyle across different lineages of ammonia-oxidizing archaea
Source: ISME J. 2025 Sep 9;19(1):wraf182. doi: 10.1093/ismejo/wraf182 (PMC12448741; doi:10.1093/ismejo/wraf182)

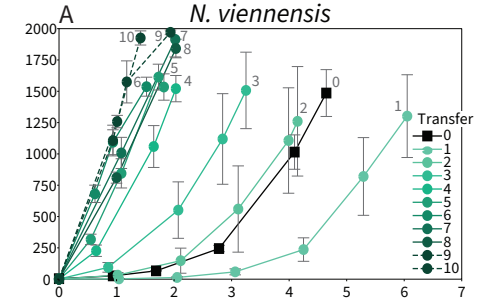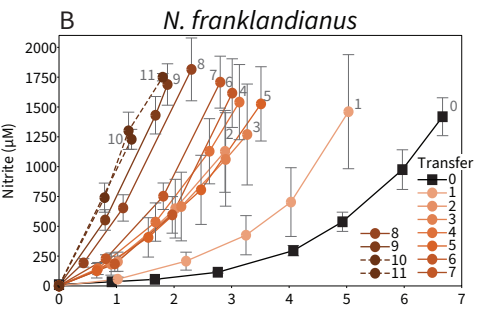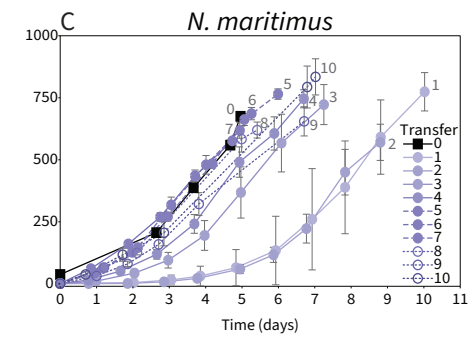

Supplement: Fig_S5_wraf182 [file fig_s5_wraf182.pdf]
