# Supplementary material for: Biofilm lifestyle across different lineages of ammonia-oxidizing archaea
Source: ISME J. 2025 Sep 9;19(1):wraf182. doi: 10.1093/ismejo/wraf182 (PMC12448741; doi:10.1093/ismejo/wraf182)
Supplement: Supplementary_clean_FINAL_wraf182 [file supplementary_clean_final_wraf182.pdf]

Supplementary Material

Biofilm lifestyle across different lineages of ammonia-oxidizing archaea

Maximilian Dreer*[1], Thomas Pribasnig*[1], Logan H. Hodgskiss[#1], Zhen-Hao Luo[1], Fran Pozaric[1], Christa Schleper[#1]

* These authors contributed equally

[1] Department of Functional and Evolutionary Ecology, Archaea Biology and Ecogenomics Unit, University of Vienna, Djerassiplatz 1, 1030 Vienna, Austria

[#]Corresponding authors: christa.schleper@univie.ac.at, logan.hodgskiss@univie.ac.at

This file contains:

Dataset Descriptions

Supplementary Materials and Methods

Supplementary Table S1-2

Supplementary Figures S1-5

Dataset Descriptions

Dataset_S1_growth_rawdata_curves_quantification.xlsx
This excel file contains raw growth data in the form of nitrite measurements of all 6 strains used in this study, used for figure 1. Additionally includes raw growth data from growth on microscopy slides, and biofilm quantification according to calculated inoculation volumes.

Dataset_S2_Nviennensis.xlsx
This excel file contains all transcriptomic data for *N. viennensis*, raw counts, DeSeq output and calculated TPMs for all genes. Additionally, significantly differently expressed genes, and the Top25 differently expressed genes used in figure 4 are displayed here.

Dataset_S3_Nfranklandianus.xlsx
This excel file contains all transcriptomic data for *N. franklandianus*, raw counts, DeSeq output and calculated TPMs for all genes. Additionally, significantly differently expressed genes, and the Top25 differently expressed genes used in figure 4 are displayed here.

Dataset_S4_Nmaritimus.xlsx
This excel file contains all transcriptomic data for *N. maritimus*, raw counts, DeSeq output and calculated TPMs for all genes. Additionally, significantly differently expressed genes, and the Top25 differently expressed genes used in figure 4 are displayed here.

Dataset_S5_conserved_upreg_protein_families_Venn_diagramm.xlsx
This excel file contains the conserved protein families between *N. viennensis, N. franklandianus* and *N. maritimus* in all possible combinations, used in supplementary figure S1.

Dataset_S6_figures_discussion_data.xlsx
This excel file contains transcriptomic data from *N. viennensis, N. franklandianus* and *N. maritimus* used in figures 5&6, all conserved upregulated protein families, CAZymes, and species used for calculating protein families. Additionally includes correlation analysis of TFB Nmar_0013 and diamond hits of adhesion proteins and sialidases.

Supplementary Material and Methods

The whole-transcriptome data generated in this study have been deposited in the NCBI BioProject database under the accession project number (PRJNA1156597), Submission ID: SUB14703162 (https://www.ncbi.nlm.nih.gov/sra/PRJNA1156597).

All additional datasets generated during the current study are provided in the assocated datasets or is available in the pribasnig repository, under following link: https://github.com/pribasnig/Biofilm_NV_NF_NM .

Strains

Nitrososphaera viennensis EN76 was isolated from garden soil [1]. *Ca.* Nitrosocosmicus franklandianus C13 (hereafter referred to as *N. franklandianus*) was isolated from arable soil and kindly provided by Dr. Graeme Nicol (Ecole Centrale de Lyon) [2]. *Nitrosotalea sinensis* Nd2 was isolated from acidic paddy field soil and kindly provided by Dr. Graeme Nicol (Ecole Centrale de Lyon) [3]. *Nitrosopumilus maritimus* SCM1 was isolated from the sediment of a tropical marine aquarium and kindly provided by Dr. Martin Könneke (University of Washington, Seattle) [4]. *Nitrosopumilus piranensis* D3C and *Nitrosopumilus adriaticus* NF5 were both isolated from coastal marine surface waters and kindly provided by Dr. Barbara Bayer (University of Vienna) [5].

Culture maintenance

Cultures of the terrestrial ammonia-oxidizing archaeon (AOA) *N. viennensis*, *N. franklandianus*, and *N. sinensis* were grown in freshwater medium (FWM) buffered with HEPES or MES, whereas the marine AOA *N. maritimus*, *N. adriaticus, and N. piranensis* were grown in synthetic crenarchaeota medium (SCM) as described previously. For a detailed description of FWM and SCM refer to previously published materials and methods[4,6]. All cultures were supplemented with ammonia and grown in 30 ml polystyrene containers (Greiner Bio-One, G201170) holding 20 ml medium in the dark. AOA were incubated at and supplemented with following temperatures and ammonia concentrations: *N. maritimus*, *N. adriaticus, and N. piranensis* [1 mM ammonia, 28°C]. *N. sinensis* [0.5 mM ammonia, 35°C]. *N. viennensis*, *N. franklandianus* [2 mM ammonia, 42°C]. The pH of FWM buffered with HEPES or MES was between 7.5-7.7 and 5.0-5.3 respectively. The pH of unbuffered SCM media was between 7.3-7.5. Media were prewarmed before inoculation. *N. viennensis* and *N. franklandianus* were shaken at 80 r.p.m. to improve oxygen diffusion. *N. viennensis* was grown without the addition of vitamins and with 1 mM pyruvate to scavenge reactive oxygen species hydrogen peroxide (H_2O_2) otherwise inhibiting growth [7]. For detailed descriptions of each

species full medium and standard growth conditions refer to the above cited isolation papers and supplementary table S1. The growth of all stock cultures was followed by measuring nitrite concentrations colorimetrically via the Griess reaction as described previously [6]. Final concentrations of all media constituents are summarized in Table S1. Stock cultures were routinely checked for contamination microscopically and by spotting culture aliquots on R2A agar plates.

Growth as biofilm on microscopy slides

According to initial RNA extraction and quality control pretests of CG grown biofilms the extracted RNA concentrations of all three chosen model species of AOA, *N. viennensis, N. franklandianus*, and *N. maritimus*, were not sufficient for transcriptomic analysis. When RNA was sent for sequencing, the reported values were unexpectedly lower than the recommended thresholds. To increase biofilm biomass the provided surface area was increased by switching from CG to MS grown in larger containers, keeping the surface area to volume ratio almost identical (CG: 0.324, MS: 0.316). Pretests during which excess liquid of MS was removed using UV sterilized whatman paper showed a considerably faster increase of nitrite production for early transfers, not comparable to the CG setup (Dataset S1). The accelerated nitrite production was most likely due to the fact that the whatman paper insufficiently removed the excess liquid, visible to the naked eye, and therein contained planktonic cells. A steady increase of nitrite production over consecutive transfers was restored when carefully dipping the MS in prewarmed medium three times before transfer to fresh medium (Fig. S5, Dataset S1). The CG and MS setups displayed analogous trends regarding the increase of nitrite production with consecutive transfers as well as species specific maxima of nitrite production. Minimal differences were observed as follows: (I) *N. viennensis* MS nitrite production dropped from initial growth "0" to the first transfer "1", before steadily increasing (Dataset S1). (II) The MS species specific maxima of nitrite production of *N. viennensis* and *N. franklandianus* were slightly higher, and that of *N. maritimus* slightly lower (Dataset S1).

Inoculation volume corresponding to biofilm

To calculate the approximate % inoculation volume (volume/volume) of the biofilms of *N. viennensis*, *N. franklandianus*, and *N. maritimus* after each transfer, a standard curve was generated using eight different inoculation volumes (0.25%–20% v/v). The time required to metabolize half of the available ammonium (1000 µM for *N. viennensis* and *N. franklandianus*; 500 µM for *N. maritimus*) was determined using linear regression between the two nearest measurements. The same approach was used to calculate times required to metabolize half

of the available ammonium for each biofilm CG transfer. An exponential regression was used to fit the standard curve data ($R^2 > 0.99$) yielding a formula to calculate the biofilm inoculation volume, and therefore approximate cell numbers. All calculated inoculation volumes and calculations can be found in Dataset S1.

RNA extraction

Total RNA was extracted from five biological replicates of frozen microscopy slides (MS) containing *N. viennensis*, *N. franklandianus*, and *N. maritimus* biofilms, and five planktonic batch controls, grown in 125 mL species-specific medium in 250 mL Schott bottle flasks (Schott). Biofilm samples were harvested when species' accumulated nitrite in a consistent manner after multiple transfers of microscopy slides (*N.viennensis*, transfer 9; *N. franklandianus*, transfer 10) or reached maximal nitrite production levels (*N. maritimus*, transfer 7) (Dataset S1, Figure S5). Cells were harvested via filtration (MCE Membrane Filter, 0.2 µm pore size, 47 mm, Merck) during mid exponential growth. Following the filtration step, filters were inserted into 15 mL falcon tubes (Fisher Scientific), immediately frozen on dry ice and stored at -70°C. RNA of *N. viennensis* and *N. maritimus* samples was extracted from filters and MS using the mirVana miRNA Isolation Kit (Thermo Fisher) according to the manufacturer's instructions. The lysis procedure was adjusted for *N. franklandianus* as follows. After resuspending cells in the lysis/binding buffer, *N. franklandianus* cells were transferred to a screw tube containing lysis matrix B (0.1 mm silica spheres, MP Biomedicals) and mechanically homogenized three times using a FastPrep-24 Classic Instrument (MP Biomedicals) at 4.0 m/s, 25 s, cooling samples on ice for 5 minutes after each run. The RNA extraction was continued as described in the manual, finally eluting RNA in 50-100 µl of DEPC. Concentrations and possible contaminations were measured by Nanodrop (NanoPhotometer N60, Implen). If necessary, phenol was removed in an additional cleanup step as follows. The volume of the samples was adjusted to 300 µl with DEPC. After adding 30 µl 3 M Na-Acetate and 1 µl Glycogen, samples were vortexed, 550 µl 100% EtOh was added and samples incubated on ice for 1 hour. RNA was pelleted by centrifugation at 11 kxg, 20 min, 4°C, liquid discarded and RNA washed with 70% EtOH. After removing the liquid and air-drying the pellet for 10 min, RNA was resuspended in 50-100 µl of DEPC. Finally RNA was DNAse digested (with TURBO DNase (Thermo Fisher) for 1h. To exclude any traces of DNA were still left in the samples, a PCR amplification was done using *mco4_b* (NVIE_019250) primer [6], or *amoA* primer [8] as previously described.

Transcriptomic analysis

RNA was submitted to the Vienna Biocenter Facility (VBC) for NovaSeq S4 PE150 XP (Illumina) sequencing with a species specific rRNA depletion step. Raw reads were downloaded from VBC, checked using md5sum and are available online under the link provided above. FastQC v.0.12.1[9] and multiQC v.1.25[10] were used to initially analyze the data. Reads were trimmed using fastp v.0.23.4[11] with following specific settings: --trim_front1 12 --trim_poly_g --detect_adapter_for_pe --average_qual 30 --length_required 30. rRNA reads were sorted out with SortmeRNA[12], and remaining mRNA reads were mapped to the specific genomes using HISAT2 v.2.2.1[13] and counted using featureCounts[14]. Raw counts are available for all datasets. All further analyses were conducted in R-Studio version 2021.09.1+372[15], and samples from biofilms were compared against planktonic samples as controls. All scripts are available on github under: https://github.com/pribasnig/Biofilm_NV_NF_NM. Unnormalized counts were analyzed for differential expression using DESeq2 employing the default settings[16]. The Benjamini Hochberg DESeq2 default step was used to control for false discovery rate (FDR). Genes with an adjusted P value <0.01 and a $\log_2$-FC in biofilms of ≥ 1.0 or ≤ -1.0 were considered significantly up/downregulated. For principal component analysis, data was normalized using the rlog function in DESeq2. A PCA plot was then produced from the normalized data using the R packages ggplot2 [17] and ggrepel [18]. Conditions were tested for significant differences using PERMANOVA analysis from the R package vegan [19]. TPM values were calculated by dividing the read counts by the length of each gene in kilobases, yielding reads per kilobase (RPK). All RPK values per sample were summed and divided by 1 000000 giving a per-million scaling factor. Finally RPK values were divided by the scaling factor to yield transcripts per million (TPM). Log_2 of TPM was calculated to use for color coding of TPM values.

Protein family conservation

Conservation of genes between *N. viennensis*, *N. franklandianus*, and *N. maritimus* was analyzed by attributing protein families to AOA genes as previously described by [20]. Briefly, amino acid sequences of protein coding genes for selected AOA were analyzed using BLAST (v2.12.0+) in an “all sequences against all” setting with an *E* value set to 10^{-5}. Sequences with hits were used for subsequent analysis. SiLiX (v1.3.0) [21] was used to assign sequences to protein families. Sequences were considered to be within the same family if they shared 35% identity (default) and the BLAST alignment covered 70% (-r 0.7) of the sequence lengths.The HiFiX program (v1.0.6) [22] with default settings was used to refine the SiLiX families. A list of AOA genomes used can be found in Dataset S1. Once assigned,

conservation of protein families between the species was compared using the package dplyr in R [23]. Based on their protein families, genes significantly upregulated in all possible combinations of the 3 species were identified (NV+NF+NM, NV+NF, NV+NM, NF+NM, NV, NF, NM). A venn diagram was produced with the R package VennDiagram [24], displaying the results. To further investigate the genes important under growth in biofilms, the top 50 genes in log2FC were further filtered to also be among the top 100 TPM BF. Genes that failed the defined cutoffs, but that were in the same genomic region and followed the same general trends of upregulation and expression as above defined genes were manually added for further analysis.

Phylogenetic analysis of multicopper oxidase genes

The candidate sequences of MCO were identified via hmmsearch v3.4 [25] (PF07731 and PF07732) against the AOA representative genomic dataset (completeness ≥ 95% and contamination < 5%; see Dataset X), and then aligned with MAFFT v7.526 [26]. Poorly aligned regions with ≥ 95% gaps were filtered with TrimalAL v1.5 [27] and the maximum-likelihood phylogeny for the MCO alignment was inferred using the IQ-TREE v2.3.4 [28] with 1000 UFBoot replicates. The atypical sequences were removed manually. All trees were uploaded to iTOL for visualization. Multicopper oxidases were assigned to different types (MCO1, MCO2, MCO3, MCO4a, MCO4b, and NirK) based on previous classification of MCOs [29,30]. MCO type and presence/absence was incorporated into the subsequent genomic analysis (see below).

Phylogenomic analysis and AOA-wide genomic analysis

The 53 archaeal-specific concatenated phylogenetically informative markers identified with GDTB-Tk v2.4.0 [31] were chosen to generate phylogenomic trees of AOA. Only the representative genomes within the families *Nitrosocaldaceae*, *Nitrosopumilaceae* and, *Nitrososphaeraceae* in the GTDB database with completeness ≥95% and contamination ≤5% were retained as the final AOA genomic dataset. Additionally, *Nitrosomirales* genomes [32] and high-quality AOA genomes of the family *Nitrososphaeraceae* [33] were supplemented to provide a broader view. The final AOA representative genomic dataset was obtained via de-replicating all genomes at 95% ANI (species level) with dRep v3.5.0 [34]. Poorly aligned regions were removed using TrimAL v1.4 with the parameters "-gt 0.95 –cons 50". The maximum-likelihood phylogeny for the concatenated alignment was inferred using the IQ-TREE v2.3.4 with 1000 UFBoot replicates. The best-fitting protein model, Q.insect+F+R8, was determined using ModelFinder.

The candidate sequences of CopCD were identified via hmmsearch v3.4 (PF05425; CopD PFAM, also covers CopC sequences) against the AOA representative genomic dataset to identify presence/absence of CopC and/or CopD.

As no defined PFAMs were found for sialidases and adhesion proteins, the candidate sequences of sialidases (WP_144239548.1, WP_172602238.1, WP_075053675.1, WP_144239600.1, WP_134484667.1, WP_172602240.1, and, WP_134484081.1) and adhesion proteins (WP_158435009.1, WP_012214839.1, and, WP_160289370.1) were identified via query of the AOA representative genomic dataset against the representative sequences from *N. viennensis*, *N. franklandianus*, and, *N. maritimus* from NCBI using diamond[35] with an e-value of 1e-5.

Supplementary tables and figures

Table S1: Growth conditions of AOA species used in this study.

Media composition of *N. viennensis, N. franklandianus, N. sinensis, N. maritimus, N. adriaticus, and N. piranensis*

Final concentration (mM)

Salts [autoclaved]

	NaCl	MgCl2 6H2O	CaCl2 2H2O	KCl	NaHCO3	MgSO4 7H2O	KH2PO4
N. viennensis	17.0	1.90	6.8	6.7	2	X	1.14
N. franklandianus	17.0	1.90	6.8	6.7	2	X	1.14
N. sinensis	17.0	1.90	6.8	6.7	2	X	1.14
N. maritimus, N. adriaticus & N. piranensis	444.9	24.59	10.2	X	1	20.28	*

Trace Elements [filtered]

	Non-chelated trace elements									FeNaEDTA solution
	HCl	H3BO3	MnCL2 4H2O	CoCL2 6H2O	NiCl2 6H2O	ZnSO4 7H2O	Na2MoO4 2H2O	(NH4)6 Mo7O24 4H2O	CuCl H2O	
N. viennensis	0.1	5*10-4	5*10-4	8*10-4	1*10-4	5*10-4	1.5*10-4	X	1.4*10-5	0.75
N. franklandianus	0.1	5*10-4	5*10-4	8*10-4	1*10-4	5*10-4	1.5*10-4	X	1.4*10-5	0.75
N. sinensis	12.5	5*10-4	5*10-4	8*10-4	1*10-4	5*10-4	X	2.9*10-5	1.4*10-5	0.75
N. maritimus, N. adriaticus & N. piranensis	0.1	5*10-4	5*10-4	8*10-4	1*10-4	5*10-4	1.5*10-4	X	1.4*10-5	0.75

	Vitamins [filtered]										
		Vitamin solution									
		Biotin	Folic acid	Pyrodoxine HCl	Thiamine HCl	Riboflavin	Nicotinic acid	DL Panthothenic acid	p-aminobenzoic acid	Choline chloride	Vitamin B12
N. viennensis	X										
N. franklandianus	+	8*10-5	5*10-5	4.8*10-4	1.4*10-4	1.3*10-4	4*10-4	2*10-4	3.6*10-4	1.4*10-3	7.3*10-9
N. sinensis	X										
N. maritimus, N. adriaticus & N. piranensis	X										

	Nutrients [filtered]	Buffer (pH) [filtered]		Antibiotics [filtered]	Other [filtered]	
	NH4Cl	HEPES (7.5)	MES (5)	Kanamycin	Na-pyruvate	KH2PO4*
N. viennensis	2	10	X	8.5*10-2	1	X
N. franklandianus	2	10	X	8.5*10-2	X	X
N. sinensis	0.5	X	10	X	X	X
N. maritimus, N. adriaticus & N. piranensis	1	X	X	X	X	2.29

Growth conditions & transfer criteria of *N. viennensis, N. franklandianus, N. sinensis, N. maritimus, N. adriaticus, and N. piranensis*

	Temperature (°C)	Shaking (rpm)	Stock inoculation volume (%v/v)	Nitrite at transfer (mM)
N. viennensis	42	80	0.25	1.20 - 1.80
N. franklandianus	42	80	1.00	1.20 - 1.80
N. sinensis	35	X	2.00	0.15 - 0.25
N. maritimus, N. adriaticus & N. piranensis	28	X	5.00	0.50 - 0.80

* KH2PO4 added seperately to marine AOA medium

X not added

Table S2: Biomass accumulation ratios* per transfer per organism

	Biomass accumulation ratio											
Transfer	1	2	3	4	5	6	7	8	9	10	11	12
N. viennensis	0.61	0.79	0.90	0.95	1.27	1.42	1.68	1.89	2.32	2.20	2.53	
N. franklandianus	0.93	1.09	1.18	1.73	2.92	3.44	4.14	3.83	4.33			
N. maritimus	0.80	1.02	0.98	1.30	1.68	1.38	1.25	1.22	1.28			
N. adriaticus	0.83	1.07	1.22	1.25	1.79	2.01	2.15	1.73	1.53	1.40	1.15	1.03
N. piranensis	0.46	0.43	0.44	0.53	0.54	0.64	0.72					

*The here defined biomass accumulation ratio (BAR) was calculated to compare biofilm forming capabilities of strains with differing generation times. For this, the time required to produce 500 µM of nitrite was determined for both, planktonic 5% inoculation volume cultures and all CG transfers and termed standard time (ST) and biofilm time (BT) respectively. The BAR was then calculated by dividing the ST by the BT for each transfer, expressed as BAR = ST/BTx, where x represents the transfer number**.**

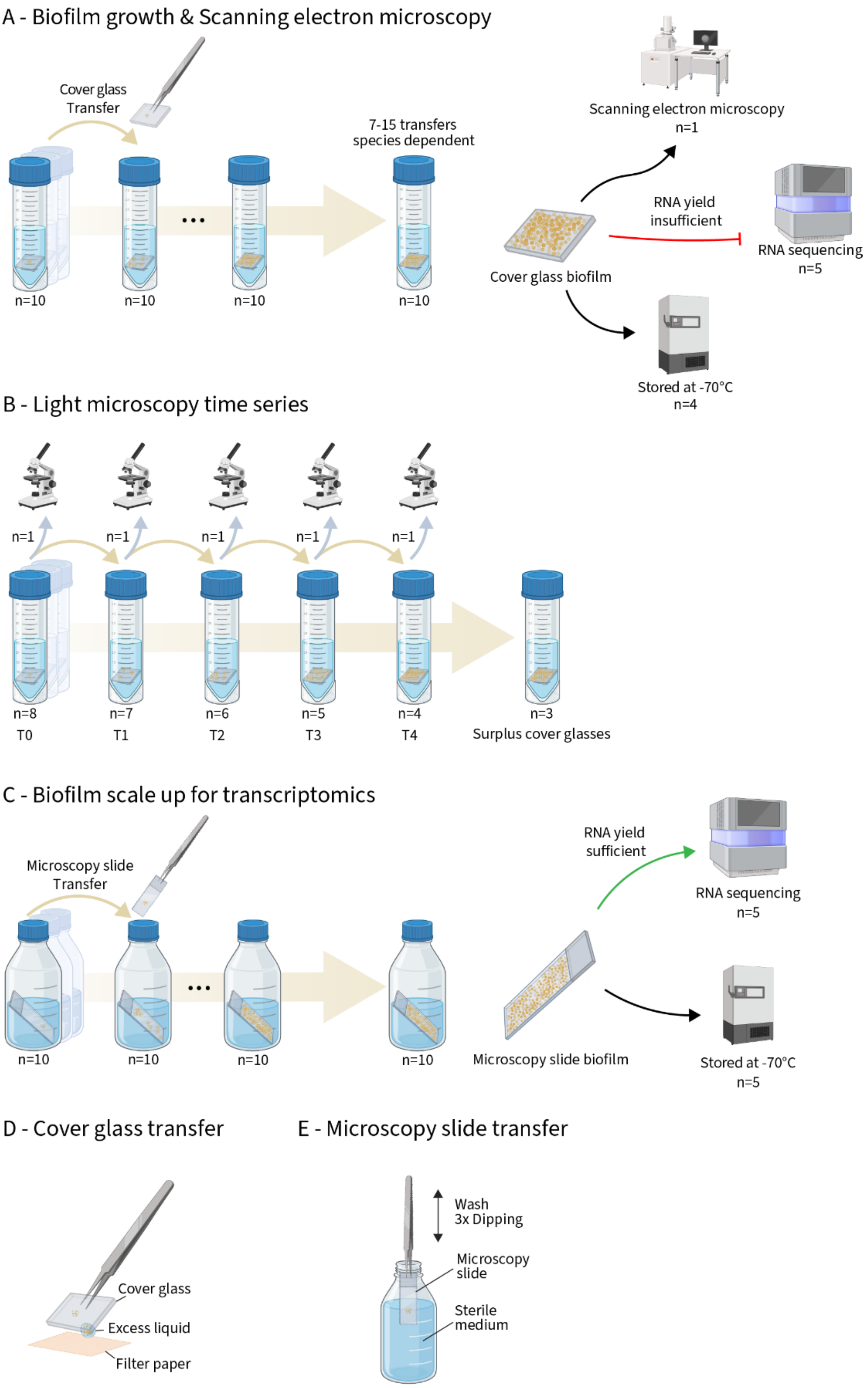
A - Biofilm growth & Scanning electron microscopy
Cover glass
Transfer
7-15 transfers
species dependent
n=10
n=10
n=10
n=10
Scanning electron microscopy
n=1
RNA yield
insufficient
RNA sequencing
n=5
Cover glass biofilm
Stored at -70°C
n=4
B - Light microscopy time series
n=1
n=1
n=1
n=1
n=1
n=8
T0
n=7
T1
n=6
T2
n=5
T3
n=4
T4
n=3
Surplus cover glasses
C - Biofilm scale up for transcriptomics
Microscopy slide
Transfer
RNA yield
sufficient
RNA sequencing
n=5
n=10
n=10
n=10
n=10
Microscopy slide biofilm
Stored at -70°C
n=5
D - Cover glass transfer
Cover glass
Excess liquid
Filter paper
E - Microscopy slide transfer
Wash
3x Dipping
Microscopy
slide
Sterile
medium

Supplementary Figure S1: Scheme of experimental methodologies (A) Biofilm growth on cover glass (B) Time series of biofilm formation on cover glass (C) Biofilm growth on microscopy slides for biomass scale up (D) Cover glass transfer using sterile whatman paper (E) Microscopy slide transfer via dipping in prewarmed medium. Created in BioRender. Dreer, M. (2025) https://BioRender.com/96w3lzd.

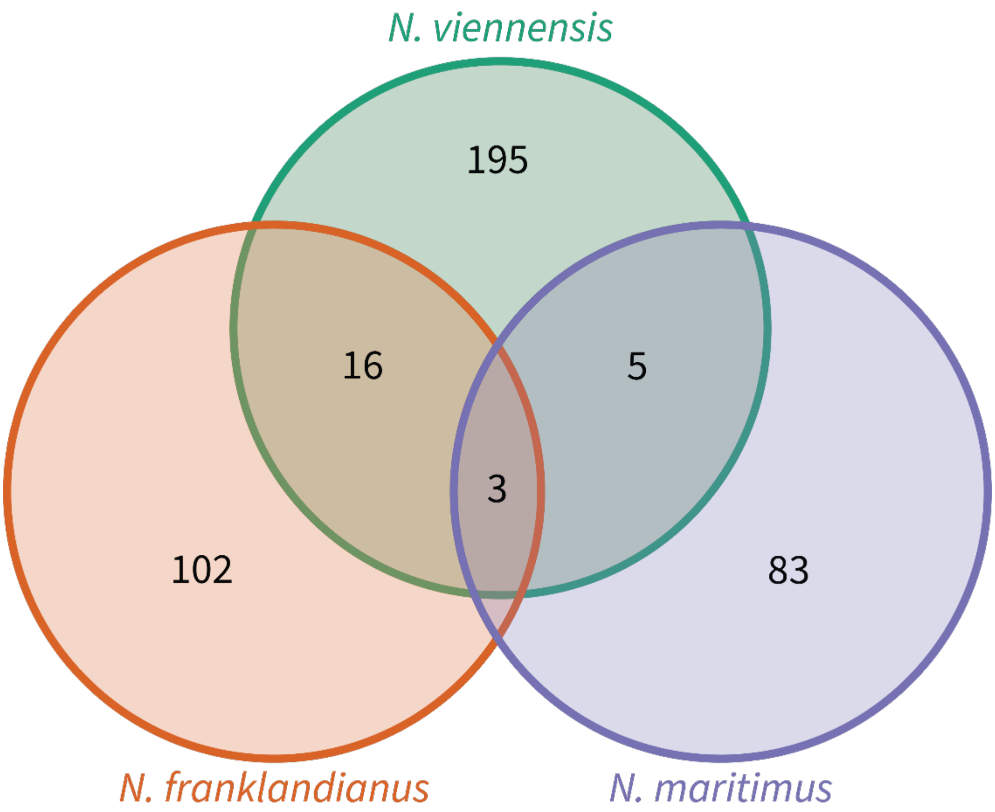

Supplementary Figure S2: Venn Diagram of shared protein families of significantly upregulated genes in biofilms (log2FC >1, padj <0.001). The Venn Diagram shows shared or unique protein families between *N. viennensis, N. franklandianus*, and *N. maritimus* in all possible combinations.

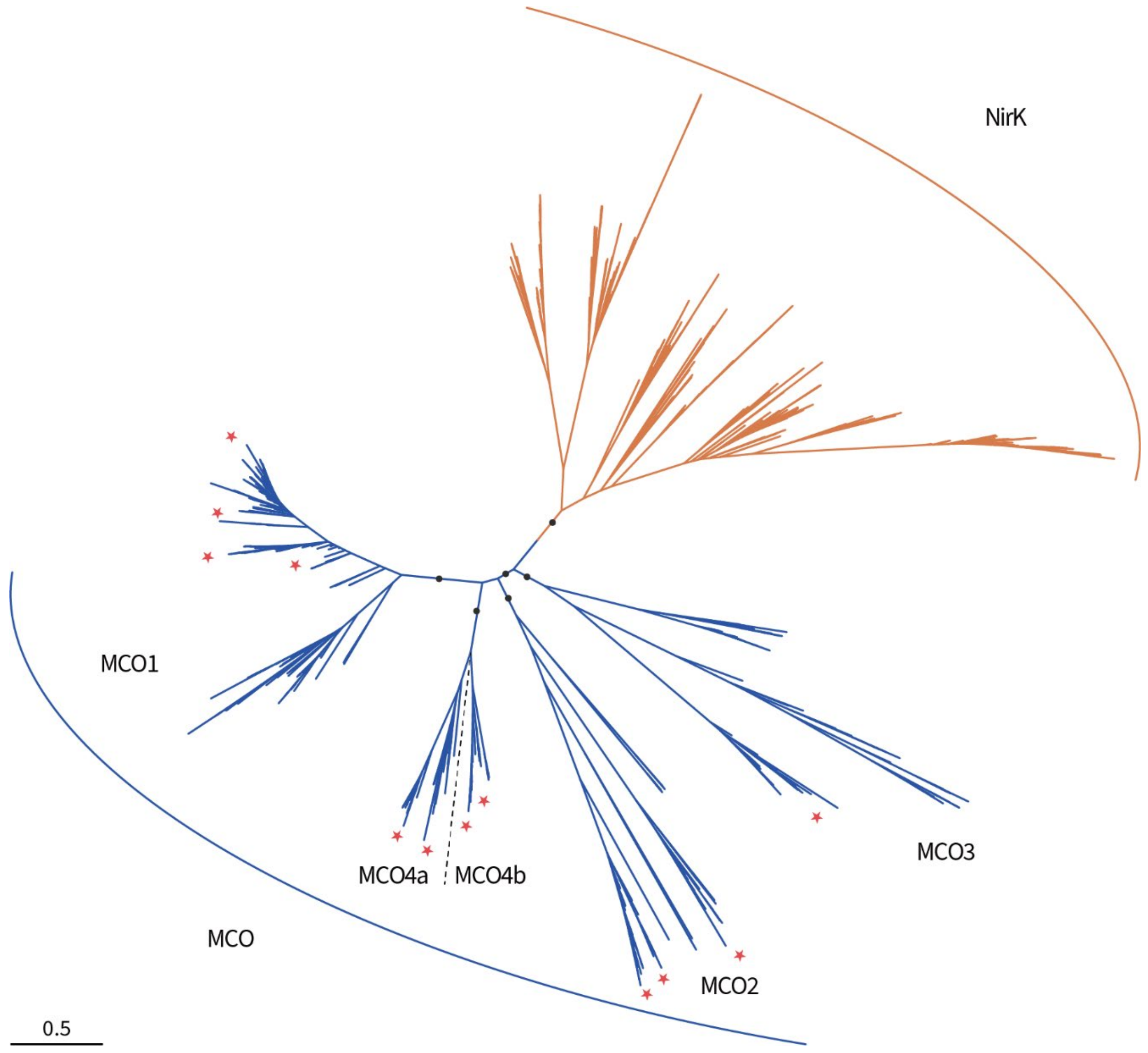

Supplementary Figure S3: The full unrooted tree for NirK and multicopper oxidases (MCO1, MCO2, MCO3, MCO4a, and MCO4b). NirK and MCO sequences are shown in orange and blue respectively. The placements of MCOs of organisms included in this study are marked with red asterisks. Nodes with ultrafast boot strap ≥ 80% are indicated as solid circles and the scale bar indicates 50% sequence divergence.

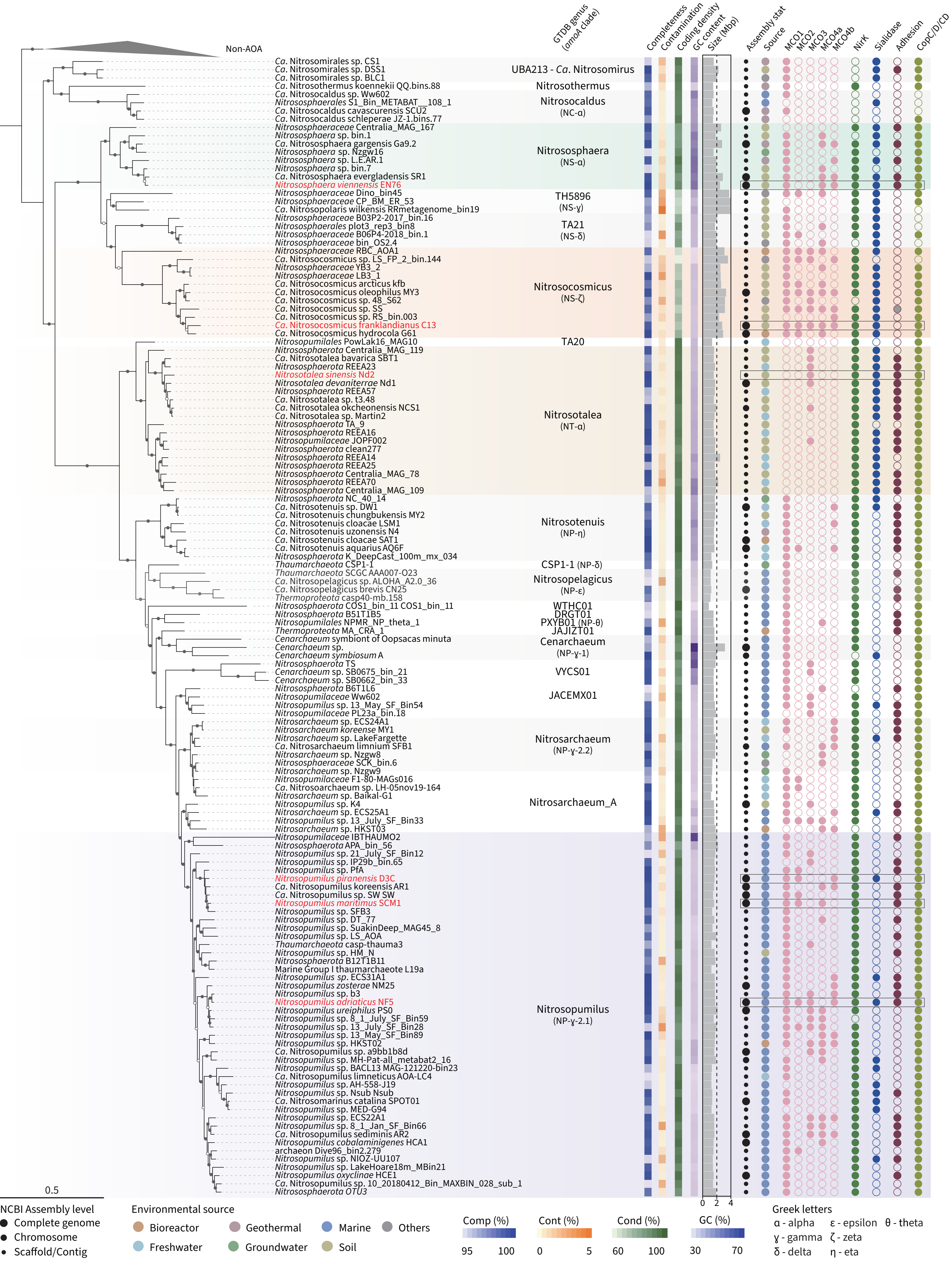

Non-AOA
GTDB genus (amoA clade)
Completeness
Contamination
Coding density
GC content
Size (Mbp)
Assembly stat
Source
MCO1
MCO2
MCO3
MCO4a
MCO4b
NirK
Sialidase
Adhesion
CopC/D/CD
Ca. Nitrosomirales sp. CS1
Ca. Nitrosomirales sp. DSS1
Ca. Nitrosomirales sp. BLC1
Ca. Nitrosothermus koennekii QQ.bins.88
Ca. Nitrosocaldus sp. Ww602
Nitrososphaerales S1_Bin_METABAT__108_1
Ca. Nitrosocaldus cavascurensis SCU2
Ca. Nitrosocaldus schleperae JZ-1.bins.77
Nitrososphaeraceae Centralia_MAG_167
Nitrososphaera sp. bin.1
Ca. Nitrososphaera gargensis Ga9.2
Nitrososphaera sp. Nzgw16
Nitrososphaera sp. L.E.AR.1
Nitrososphaera sp. bin.7
Ca. Nitrososphaera evergladensis SR1
Nitrososphaera viennensis EN76
Nitrososphaeraceae Dino_bin45
Nitrososphaeraceae CP_BM_ER_53
Ca. Nitrosopolaris wilkensis RRmetagenome_bin19
Nitrososphaeraceae B03P2-2017_bin.16
Nitrososphaerales plot3_rep3_bin8
Nitrososphaeraceae B06P4-2018_bin.1
Nitrososphaeraceae bin_OS2.4
Nitrososphaeraceae RBC_AOA1
Ca. Nitrosocosmicus sp. LS_FP_2_bin.144
Nitrososphaeraceae YB3_2
Nitrososphaeraceae LB3_1
Ca. Nitrosocosmicus arcticus kfb
Ca. Nitrosocosmicus oleophilus MY3
Ca. Nitrosocosmicus sp. 48_S62
Ca. Nitrosocosmicus sp. SS
Ca. Nitrosocosmicus sp. RS_bin.003
Ca. Nitrosocosmicus franklandianus C13
Ca. Nitrosocosmicus hydrocola G61
Nitrosopumilales PowLak16_MAG10
Nitrososphaerota Centralia_MAG_119
Ca. Nitrosotalea bavarica SBT1
Nitrososphaerota REEA23
Nitrosotalea sinensis Nd2
Nitrosotalea devaniterrae Nd1
Nitrososphaerota REEA57
Ca. Nitrosotalea sp. t3.48
Ca. Nitrosotalea okcheonensis NCS1
Ca. Nitrosotalea sp. Martin2
Nitrososphaerota TA_9
Nitrososphaerota REEA16
Nitrosopumilaceae JOPF002
Nitrososphaerota clean277
Nitrososphaerota REEA14
Nitrososphaerota REEA25
Nitrososphaerota Centralia_MAG_78
Nitrososphaerota REEA70
Nitrososphaerota Centralia_MAG_109
Nitrososphaerota NC_40_14
Ca. Nitrosotenuis sp. DW1
Ca. Nitrosotenuis chungbukensis MY2
Ca. Nitrosotenuis cloacae LSM1
Ca. Nitrosotenuis uzonensis N4
Ca. Nitrosotenuis cloacae SAT1
Ca. Nitrosotenuis aquarius AQ6F
Nitrososphaerota K_DeepCast_100m_mx_034
Thaumarchaeota CSP1-1
Thaumarchaeota SCGC AAA007-O23
Ca. Nitrosopelagicus sp. ALOHA_A2.0_36
Ca. Nitrosopelagicus brevis CN25
Thermoproteota casp40-mb.158
Nitrososphaerota COS1_bin_11 COS1_bin_11
Nitrososphaerota B51T1B5
Nitrosopumilales NPMR_NP_theta_1
Thermoproteota MA_CRA_1
Cenarchaeum symbiont of Oopsacas minuta
Cenarchaeum sp.
Cenarchaeum symbiosum A
Nitrososphaerota TS
Cenarchaeum sp. SB0675_bin_21
Cenarchaeum sp. SB0662_bin_33
Nitrososphaerota B6T1L6
Nitrosopumilaceae Ww602
Nitrosopumilus sp. 13_May_SF_Bin54
Nitrosopumilaceae PL23a_bin.18
Nitrosarchaeum sp. ECS24A1
Nitrosarchaeum koreense MY1
Nitrosarchaeum sp. LakeFargette
Ca. Nitrosarchaeum limnium SFB1
Nitrosarchaeum sp. Nzgw8
Nitrososphaeraceae SCK_bin.6
Nitrosarchaeum sp. Nzgw9
Nitrosopumilaceae F1-80-MAGs016
Ca. Nitrosoarchaeum sp. LH-05nov19-164
Nitrosarchaeum sp. Baikal-G1
Nitrosopumilus sp. K4
Nitrosarchaeum sp. ECS25A1
Nitrosopumilus sp. 13_July_SF_Bin33
Nitrosarchaeum sp. HKST03
Nitrosopumilaceae IBTHAUMO2
Nitrososphaerota APA_bin_56
Nitrosopumilus sp. 21_July_SF_Bin12
Nitrosopumilus sp. IP29b_bin.65
Nitrosopumilus sp. PfA
Nitrosopumilus piranensis D3C
Ca. Nitrosopumilus koreensis AR1
Ca. Nitrosopumilus sp. SW SW
Nitrosopumilus maritimus SCM1
Nitrosopumilus sp. SFB3
Nitrosopumilus sp. DT_77
Nitrosopumilus sp. SuakinDeep_MAG45_8
Nitrosopumilus sp. LS_AOA
Thaumarchaeota casp-thauma3
Nitrosopumilus sp. HM_N
Nitrososphaerota B12T1B11
Marine Group I thaumarchaeote L19a
Nitrosopumilus sp. ECS31A1
Nitrosopumilus zosterae NM25
Nitrosopumilus sp. b3
Nitrosopumilus adriaticus NF5
Nitrosopumilus ureiphilus PS0
Nitrosopumilus sp. 8_1_July_SF_Bin59
Nitrosopumilus sp. 13_July_SF_Bin28
Nitrosopumilus sp. 13_May_SF_Bin89
Nitrosopumilus sp. HKST02
Ca. Nitrosopumilus sp. a9bb1b8d
Nitrosopumilus sp. MH-Pat-all_metabat2_16
Nitrosopumilus sp. BACL13 MAG-121220-bin23
Ca. Nitrosopumilus limneticus AOA-LC4
Nitrosopumilus sp. AH-558-J19
Nitrosopumilus sp. Nsub Nsub
Ca. Nitrosomarinus catalina SPOT01
Nitrosopumilus sp. MED-G94
Nitrosopumilus sp. ECS22A1
Nitrosopumilus sp. 8_1_Jan_SF_Bin66
Ca. Nitrosopumilus sediminis AR2
Nitrosopumilus cobalaminigenes HCA1
archaeon Dive96_bin2.279
Nitrosopumilus sp. NIOZ-UU107
Nitrosopumilus sp. LakeHoare18m_MBin21
Nitrosopumilus oxyclinae HCE1
Ca. Nitrosopumilus sp. 10_20180412_Bin_MAXBIN_028_sub_1
Nitrososphaerota OTU3
UBA213 - Ca. Nitrosomirus
Nitrosothermus
Nitrosocaldus
(NC-α)
Nitrososphaera
(NS-α)
TH5896
(NS-γ)
TA21
(NS-δ)
Nitrosocosmicus
(NS-ζ)
TA20
Nitrosotalea
(NT-α)
Nitrosotenuis
(NP-η)
CSP1-1 (NP-δ)
Nitrosopelagicus
(NP-ε)
WTHC01
DRGT01
PXYB01 (NP-θ)
JAJIZT01
Cenarchaeum
(NP-γ-1)
VYCS01
JACEMX01
Nitrosarchaeum
(NP-γ-2.2)
Nitrosarchaeum_A
Nitrosopumilus
(NP-γ-2.1)
0.5
0 2 4
NCBI Assembly level
Complete genome
Chromosome
Scaffold/Contig
Environmental source
Bioreactor
Geothermal
Marine
Others
Freshwater
Groundwater
Soil
Comp (%)
95 100
Cont (%)
0 5
Cond (%)
60 100
GC (%)
30 70
Greek letters
α - alpha
ε - epsilon
θ - theta
γ - gamma
ζ - zeta
δ - delta
η - eta

Supplementary Figure S4: The overall distribution of genes of interest in AOA. The phylogenomic tree is the non-collapsed version of the tree shown in Fig. 7. Nodes with ultrafast bootstrap value ≥ 60% were indicated as solid circles. Strains involved in this study are marked with red. The scale bar in the bottom left corner indicates 50% sequence divergence. The full (empty) Circles represent the presence (absence) of genes. Black circles represent different assembly levels according to NCBI. In brief, "complete genomes" are closed and contain no gaps, "chromosomes" are not closed and might still contain gaps whereas "scaffolds/contigs" are fragmented and are sure to contain gaps. A grey filled circle indicates an adhesion protein hit that is annotated as a PQQ dehydrogenase and not assumed to be an adhesion protein (Dataset_S6). MCO, multicopper oxidase.

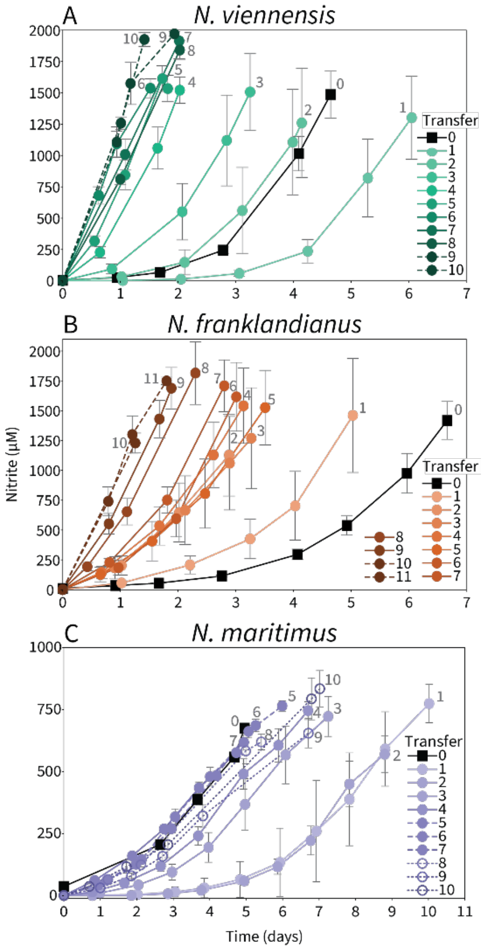

Supplementary Figure S5: Biofilm formation of ammonia-oxidizing archaea on microscopy slides (MS). Nitrite production of (A) *Nitrososphaera viennensis*, (B) *Nitrosocosmicus franklandianus*, (C) *Nitrosopumilus maritimus* grown as biofilm on MS. The raw data of all transfers can be found in Dataset S1. Initial planktonic growth of cells in the presence of CG (black lines), continuous transfers of CG (colored lines), and maximum nitrite production (colored dashed lines) are shown. Drops in nitrite production between transfers were observed for N. maritimus (C, dotted lines, empty circles). C Increasing numbers of transfers are indicated by numbers next to lines and darkening color gradients. Nitrite measurements show averages of eight biological replicates. Error bars depict the standard deviation.

References

1. Tourna M, Stieglmeier M, Spang A *et al. Nitrososphaera viennensis*, an ammonia oxidizing archaeon from soil. *Proc Natl Acad Sci U S A* 2011;**108**:8420–5.

2. Lehtovirta-Morley LE, Ross J, Hink L *et al.* Isolation of "*Candidatus* Nitrosocosmicus franklandus", a novel ureolytic soil archaeal ammonia oxidiser with tolerance to high ammonia concentration. *FEMS Microbiol Ecol* 2016;**92**:fiw057.

3. Lehtovirta-Morley LE, Ge C, Ross J *et al.* Characterisation of terrestrial acidophilic archaeal ammonia oxidisers and their inhibition and stimulation by organic compounds. *FEMS Microbiol Ecol* 2014;**89**:542–52.

4. Könneke M, Bernhard AE, De La Torre JR *et al.* Isolation of an autotrophic ammonia-oxidizing marine archaeon. *Nature* 2005;**437**:543–6.

5. Bayer B, Vojvoda J, Reinthaler T *et al. Nitrosopumilus adriaticus* sp. nov. and *Nitrosopumilus piranensis* sp. nov., two ammonia-oxidizing archaea from the adriatic sea and members of the class *Nitrososphaeria*. *Int J Syst Evol Microbiol* 2019;**69**:1892–902.

6. Reyes C, Hodgskiss LH, Baars O *et al.* Copper limiting threshold in the terrestrial ammonia oxidizing archaeon *Nitrososphaera viennensis*. *Research in Microbiology* 2020;**171**:134–42.

7. Kim J-G, Park S-J, Sinninghe Damsté JS *et al.* Hydrogen peroxide detoxification is a key mechanism for growth of ammonia-oxidizing archaea. *Proc Natl Acad Sci USA* 2016;**113**:7888–93.

8. Alves RJE, Minh BQ, Urich T *et al.* Unifying the global phylogeny and environmental distribution of ammonia-oxidising archaea based on amoA genes. *Nat Commun* 2018;**9**:1517.

9. Andrews S. FastQC a quality control tool for high throughput sequence data [Online]. Available online at: http://www.bioinformatics.babraham.ac.uk/projects/fastqc/. 2010.

10. Ewels P, Magnusson M, Lundin S *et al.* MultiQC: summarize analysis results for multiple tools and samples in a single report. *Bioinformatics* 2016;**32**:3047–8.

11. Chen S, Zhou Y, Chen Y *et al.* fastp: an ultra-fast all-in-one FASTQ preprocessor. *Bioinformatics* 2018;**34**:i884–90.

12. Schmieder R, Edwards R. Quality control and preprocessing of metagenomic datasets. *Bioinformatics* 2011;**27**:863–4.

13. Kim D, Paggi JM, Park C *et al.* Graph-based genome alignment and genotyping with HISAT2 and HISAT-genotype. *Nat Biotechnol* 2019;**37**:907–15.

14. Liao Y, Smyth GK, Shi W. featureCounts: an efficient general purpose program for assigning sequence reads to genomic features. *Bioinformatics* 2014;**30**:923–30.

15. RStudio Team. RStudio: Integrated Development for R. RStudio, PBC, Boston, MA URL http://www.rstudio.com/. 2020.

16. Love MI, Huber W, Anders S. Moderated estimation of fold change and dispersion for RNA-seq data with DESeq2. *Genome Biol* 2014;**15**:550.

17. Wickham H. *Ggplot2: Elegant Graphics for Data Analysis*. 2nd ed. Cham, Switzerland: Springer International Publishing, 2016.

18. Slowikowski K. ggrepel: Automatically Position Non-Overlapping Text Labels with “ggplot2.” 2016:0.9.6.

19. Oksanen J, Simpson GL, Blanchet FG *et al.* vegan: community ecology package. 2024.

20. Abby SS, Kerou M, Schleper C. Ancestral reconstructions decipher major adaptations of ammonia-oxidizing archaea upon radiation into moderate terrestrial and marine environments. *mBio* 2020;**11**, DOI: 10.1128/mBio.02371-20.

21. Miele V, Penel S, Duret L. Ultra-fast sequence clustering from similarity networks with SiLiX. *BMC Bioinformatics* 2011;**12**:116.

22. Miele V, Penel S, Daubin V *et al.* High-quality sequence clustering guided by network topology and multiple alignment likelihood. *Bioinformatics* 2012;**28**:1078–85.

23. Wickham H, François R, Henry L *et al.* dplyr: A grammar of data manipulation. 2023.

24. Chen H, Boutros P. Chen H, Boutros PC.. VennDiagram: a package for the generation of highly-customizable Venn and Euler diagrams in R. BMC Bioinformatics 12: 35. *BMC bioinformatics* 2011;**12**:35.

25. Eddy SR. Accelerated profile HMM searches. *PLOS Computational Biology* 2011;**7**:e1002195.

26. Katoh K, Misawa K, Kuma K *et al.* MAFFT: a novel method for rapid multiple sequence alignment based on fast Fourier transform. *Nucleic Acids Research* 2002;**30**:3059–66.

27. Capella-Gutiérrez S, Silla-Martínez JM, Gabaldón T. trimAl: a tool for automated alignment trimming in large-scale phylogenetic analyses. *Bioinformatics* 2009;**25**:1972–3.

28. Nguyen L-T, Schmidt HA, von Haeseler A *et al.* IQ-TREE: A fast and effective stochastic algorithm for estimating maximum-likelihood phylogenies. *Mol Biol Evol* 2015;**32**:268–74.

29. Kerou M, Offre P, Valledor L *et al.* Proteomics and comparative genomics of *Nitrososphaera viennensis* reveal the core genome and adaptations of archaeal ammonia oxidizers. *Proc Natl Acad Sci USA* 2016;**113**:E7937–46.

30. Reyes C, Hodgskiss LH, Kerou M *et al.* Genome wide transcriptomic analysis of the soil ammonia oxidizing archaeon *Nitrososphaera viennensis* upon exposure to copper limitation. *ISME J* 2020;**14**:2659–74.

31. Parks DH, Rinke C, Chuvochina M *et al.* Recovery of nearly 8,000 metagenome-assembled genomes substantially expands the tree of life. *Nat Microbiol* 2017;**2**:1533–42.

32. Zheng Y, Wang B, Gao P *et al.* Novel order-level lineage of ammonia-oxidizing archaea widespread in marine and terrestrial environments. *ISME J* 2024;**18**:wrad002.

33. Bei Q, Reitz T, Schädler M *et al.* Metabolic potential of Nitrososphaera-associated clades. *ISME J* 2024;**18**:wrae086.

34. Olm MR, Brown CT, Brooks B *et al.* dRep: a tool for fast and accurate genomic comparisons that enables improved genome recovery from metagenomes through de-replication. *ISME J* 2017;**11**:2864–8.

35. Buchfink B, Xie C, Huson DH. Fast and sensitive protein alignment using DIAMOND. *Nat Methods* 2015;**12**:59–60.
